# Bioactive Self‐Assembled Nanoregulator Enhances Hematoma Resolution and Inhibits Neuroinflammation in the Treatment of Intracerebral Hemorrhage

**DOI:** 10.1002/advs.202408647

**Published:** 2024-11-08

**Authors:** Wenyan Yu, Chengyuan Che, Yi Yang, Yuzhen Zhao, Junjie Liu, Aibing Chen, Jinjin Shi

**Affiliations:** ^1^ School of Pharmaceutical Sciences Zhengzhou University Zhengzhou 450001 China; ^2^ Key Laboratory of Targeting Therapy and Diagnosis for Critical Diseases Zhengzhou University Zhengzhou 450001 China; ^3^ College of Chemical and Pharmaceutical Engineering Hebei University of Science and Technology Shijiazhuang 050018 China

**Keywords:** drug delivery, hematoma clearance, intracerebral hemorrhage therapy, microglia polarization, nanotechnology

## Abstract

Hematoma and secondary neuroinflammation continue to pose a significant challenge in the clinical treatment of intracerebral hemorrhage (ICH). This study describes a nanoregulator formed through the self‐assembly of Mg^2+^ and signal regulatory protein α (SIRPα) DNAzyme (SDz), aimed at enhancing hematoma resolution and inhibiting neuroinflammation in the treatment of ICH. The structure of SDz collapses in response to the acidic endo/lysosomal microenvironment of microglia, releasing Mg^2+^ and the SIRPα DNAzyme. The Mg^2+^ then acts as a cofactor to activate the SIRPα DNAzyme. By blocking the CD47‐SIRPα signaling pathway, microglia can rapidly and effectively phagocytose red blood cells (RBCs), thereby promoting the clearance of the hematoma. Simultaneously, Mg^2+^ reset the microglia to the M2 phenotype by inhibiting the MYD88/MAPK/NF‐κB signaling pathway, thereby modulating the inflammatory microenvironment of ICH. This co‐delivery and synergistic strategy resulted in a significant reduction in hematoma size, decreasing from 11.90 to 5.84 mm^3^, and promoted recovery from ICH with minimal systemic side effects. This simple yet highly effective nanoplatform, which involves complex synergistic mechanisms, proves to be effective for ICH therapy and holds great promise for introducing novel perspectives into clinical and translational approaches for ICH.

## Graphical abstract

The study presents a nanoregulator composed of Mg^2+^ and signal regulatory protein α (SIRPα) DNAzyme for treating intracerebral hemorrhage (ICH). It enhances hematoma clearance and inhibit neuroinflammation by activating the SIRPα DNAzyme in microglia, enhancing red blood cell clearance, and shifting microglia to an anti‐inflammatory state. This approach significantly reduces hematoma size, offering new insights into ICH therapy.

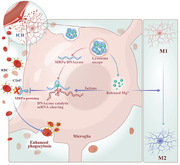

## 1 Introduction

Intracerebral hemorrhage (ICH) is a serious medical condition associated with increased rates of disability and mortality.^[^ ^1^, ^2^, ^3^ ^]^ However, unlike ischemic stroke, there have been no significant breakthroughs in the definitive treatment of ICH.^[^ ^4^, ^5^, ^6^ ^]^ The underlying pathology of ICH involves hematoma formation resulting from the rupture of acerebral blood vessels^[^ ^7^ ^]^ followed by secondary brain injury.^[^ ^8^ ^]^ Clinical data have shown that patients benefit from the near‐complete evacuation of the hematoma, underscoring the critical need for reliable hemorrhage‐evacuation techniques to ensure successful intervention in ICH.^[^ ^9^, ^10^, ^11^ ^]^ This emphasizes the pivotal role of hematoma clearance in managing this critical condition.

Currently, the primary approach used for hematoma clearance in clinical settings is surgical intervention.^[^ ^12^, ^13^, ^14^ ^]^ However, strict surgical criteria such as patient age, normal blood glucose and blood pressure levels, and the location or size of the hemorrhage limit the number of eligible patients. Moreover, patient compliance with surgery is often suboptimal. The lack of effective treatments in clinical practice has resulted in stagnation in therapeutic developments for ICH.^[^ ^15^, ^16^, ^17^ ^]^ It is particularly noteworthy that under physiological conditions, microglia play a crucial role in phagocytosis and the clearance of amyloid plaques, neurofibrillary tangles, and cell fragments in the central nervous system.^[^ ^18^, ^19^, ^20^ ^]^ Hematoma clearance by microglia represents an important endogenous mechanism in the treatment of ICH. However, CD47, expressed on red blood cells (RBCs), activates a “don't eat me” signal by binding to signal regulatory protein α (SIRPα) on the surface of microglial cells. This interaction results in immune escape and inhibits phagocytosis, thereby restricting endogenous hematoma clearance.^[^ ^21^ ^]^ Timely and effective enhancement of phagocytosis could reduce the toxic effects of the persistent presence of blood products on surrounding tissues, which is crucial for the rehabilitation of patients with ICH.^[^ ^22^, ^23^, ^24^ ^]^ For example, injection of blood in which CD47 had been knocked out resulted in faster clot resolution, reduced brain swelling, and fewer neurological deficits compared to the use of wild‐type blood.^[^ ^25^ ^]^ Furthermore, microglia are the first non‐neuronal cells to be activated in the early innate immune response to ICH.^[^ ^26^, ^27^, ^28^ ^]^ Microglial polarization and the subsequent neuroinflammatory response contribute to ICH‐induced secondary injury through the release of pro‐inflammatory cytokines, such as interleukin 12 (IL‐12) and tumor necrosis factor‐α (TNF‐α).^[^ ^29^, ^30^, ^31^ ^]^ Thus, enhancing the endogenous clearance of the hematoma by microglial cells while simultaneously controlling cellular inflammation holds promise for the treatment of cerebral hemorrhage and the improvement of prognosis.

DNAzymes, also known as deoxyribozyme, are a class of catalytic DNA molecules capable of specifically recognizing and cleaving target RNA. Consisting of target‐recognition arm sequences for substrate binding, coupled with catalytic core that operates through metal ion‐mediated activity, such as Mg^2+^, DNAzymes can specifically bind target RNA and, with the activation of metal ions, its catalytic core attacks the phosphodiester bond of the RNA substrate, thus effectively cleaving the target RNA.^[^ ^32^, ^33^, ^34^ ^]^ Emerging as powerful molecular tools, DNAzymes are increasingly valued for their promising application across various research domains. The specificity and programmability of DNAzymes make them ideal candidates for innovative therapeutic strategies in disease treatment.

**Figure 1:**
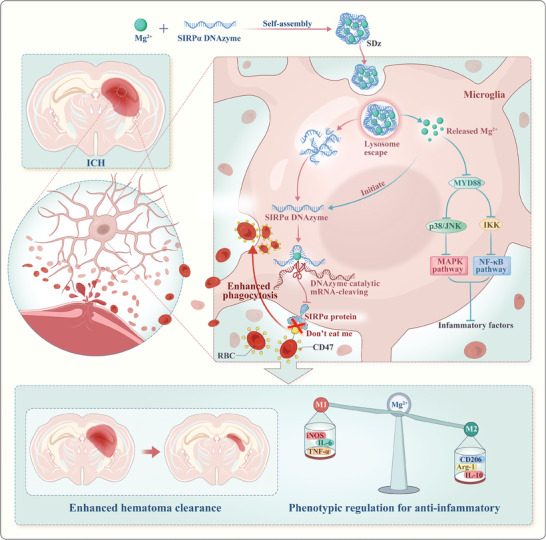
Schematic representation of the self‐assembled nanoregulator for enhancing hematoma resolution and inhibiting neuroinflammation in the treatment of intracerebral hemorrhage.

In this study, a nanoregulator (SDz) is introduced, formed through the self‐assembly of Mg^2+^ and SIRPα DNAzyme, to improve hematoma resolution and suppress neuroinflammation following ICH (**Figure** **1**). The process involves the collapse of the SDz structure in the acidic microenvironment of microglial lysosomes, leading to the release of both Mg^2+^ and the SIRPα DNAzyme. The Mg^2+^ then acts as a cofactor, activating the catalytic activity of the SIRPα DNAzyme. By blocking the CD47‐SIRPα signaling pathway, microglia can rapidly and effectively phagocytose RBCs, thereby promoting hematoma clearance. Simultaneously, Mg^2+^ regulates the microglial phenotype to alleviate neuroinflammation by inhibiting the MyD88/MAPK/NF‐κB pathway. This simple yet highly effective nanoplatform offers a synergistic mechanism for hematoma clearance and ICH therapy, holding significant promise for introducing innovative perspectives into clinical and translational approaches to ICH treatment.

## 2 Results and Discussion

### 2.1 Synthesis and Characterization of SDz

**Figure 2:**
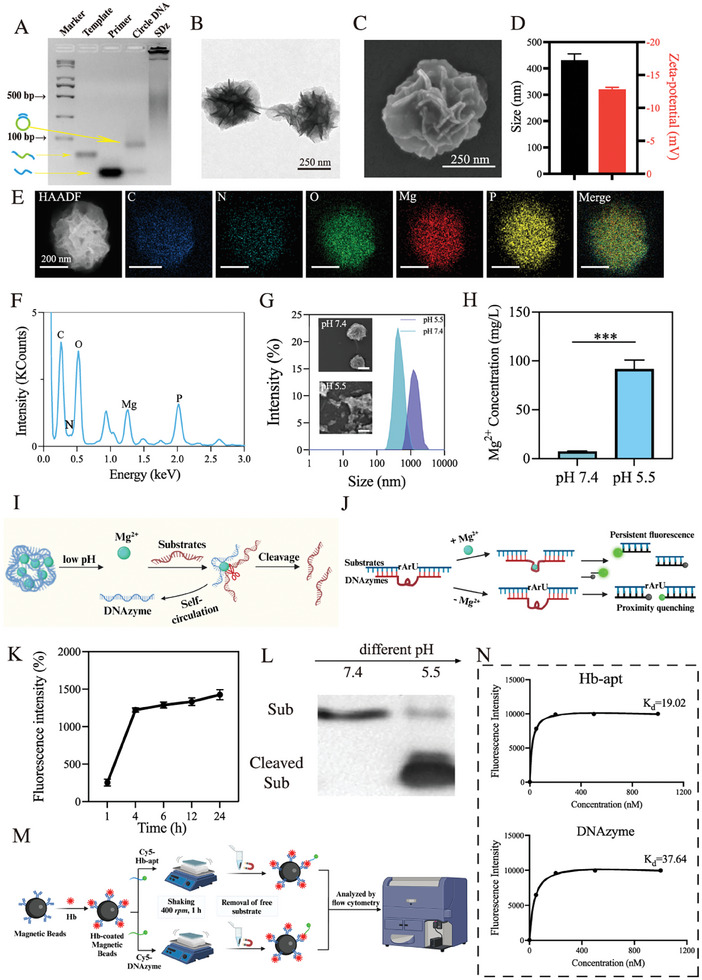
Preparation and characterization of SDz. A) Agarose gel electrophoresis of synthesized SDz. B) Representative TEM image of SDz. Scale bar: 250 nm. C) Representative SEM image of SDz. Scale bar: 250 nm. D) The particle size and zeta potential of SDz (*n* = 3). E) Representative mapping images of SDz. Scale bar: 200 nm. F) EDS spectra of SDz. G) Representative size distribution and SEM images of SDz in PBS at pH 7.4 and 5.5. The embedded figures are SEM images. Scale bar: 250 nm. H) ICP‐MS analysis of Mg^2+^ released by SDz at different pH. SDz was incubated in buffers with different pH for 24 h, after which the supernatant was analyzed by inductively coupled plasma mass spectrometry. (*n* = 3). I) Schematic diagram showing the controlled release of DNAzyme and corresponding Mg^2+^ from SDz in response to acid pH, enabling efficient cleavage of the mRNA substrate. J) The principle of the fluorescence nano assay for assessment of the DNAzyme cleavage efficiency, using fluorescence intensity for evaluation. K) Analysis of the cleavage kinetics of DNAzyme for 24 h. L) PAGE analysis of SDz‐mediated cleavage at different pH. M) Schematic diagram showing measurement of the binding affinity between the Hb aptamer (Hb‐apt) and DNAzyme with Hb using magnetic bead‐based fluorescence assay. N) Binding curves of Cy5‐labeled DNA sequence to purified Hb‐modified beads. Data are expressed as mean ± standard deviation. ***P < 0.001. Statistical analysis was performed using the unpaired two‐tailed Student's t‐test (H). SDz, SIRPα‐DNAzyme nanostructures; EDS, Energy Dispersive Spectrometer; ICP‐MS, inductively coupled plasma mass spectrometry; PAGE, polyacrylamide gel electrophoresis; Hb, hemoglobin.

In this work, a highly efficient Mg^2+^‐activated mRNA‐cleaving DNAzyme was selected to specifically recognize and silence intracellular SIRPα mRNA, thereby inhibiting protein expression (Figure S1, Supporting Information). To assess the cleaving ability, different concentrations of Mg^2+^ were incubated with the substrates. Polyacrylamide gel electrophoresis (PAGE) demonstrated that 1 mm Mg^2+^ was sufficient to activate the DNAzyme (Figure S2, Supporting Information). For efficient intracellular delivery and self‐sustained gene therapy, Mg^2+^ and the DNAzyme self‐assembled into a nanostructure (SDz) through rolling circle amplification (RCA) (sequence provided in Table S1, Supporting Information). As shown in **Figure** **2**A, due to its high relative molecular weight, SDz appeared as a stable accumulation near the sample loading position, confirming the successful synthesis of SDz. In contrast, the DNA template strand, primer strand, and cyclized products appeared near the bottom of the gel due to their lower molecular weight. The structure of SDz was further examined using transmission electron microscopy (TEM) and scanning electron microscopy (SEM) (Figure 2B,C). Dynamic light scattering (DLS) revealed that the particle size of SDz was ≈425 nm (Figures 2D and S3, Supporting Information), consistent with the SEM and TEM results, while the zeta potential of SDz was ≈−13.4 mV. In addition, elemental mapping and energy‐dispersive X‐ray spectroscopy (EDS) analysis of SDz (Figure 2E,F) revealed that SDz primarily consisted of DNA and magnesium pyrophosphate, consistent with previously reported findings.^[^ ^35^ ^]^ These results confirmed the successful preparation of SDz nanoparticles.

Magnesium pyrophosphate is known to be sensitive to acidic environments,^[^ ^36^ ^]^ which can trigger the dissociation of SDz and the release of Mg^2+^/DNAzyme. SEM images showed that the SDz structure completely collapsed in acidic solutions (Figure 2G, inset). DLS results further confirmed the disintegration of SDz in response to acidic conditions (Figure 2G). To further evaluate the dissolution of the SDz magnesium pyrophosphate core, its ability to release magnesium at different pH levels (pH 7.4 and pH 5.5) was assessed using inductively coupled plasma mass spectrometry (ICP‐MS). As shown in Figure 2H, the release of Mg^2+^ was negatively correlated with pH, indicating that acidic environments facilitated the dissociation of SDz. This also suggested the potential for acid‐driven lysosomal escape of SDz for gene silencing in cells. To verify the cleavage of SDz in vitro, a DNA nanostructure lacking DNAzyme cleavage (cDz) was constructed as a control group. SEM and TEM images confirmed the successful synthesis and spherical structure of cDz (Figure S4, Supporting Information).

Additionally, the potential of the Mg^2^⁺ released in acidic environments to serve as sufficient DNAzyme cofactors for biocatalysis was analyzed (Figure 2I). This was initially assessed using fluorescence analysis and PAGE to evaluate the DNAzyme's catalytic capability in the presence of Mg^2+^. Real‐time monitoring of shear force was conducted using fluorescence resonance energy transfer (FRET), as illustrated in Figure 2J. As expected, the fluorescence intensity increased over time, indicating robust, time‐dependent cleavage of the substrate by the DNAzyme when incubated with Mg^2+^ (Figure 2K). PAGE results further confirmed that the Mg^2+^ released from SDz in an acidic environment was fully sufficient to initiate DNAzyme activity (Figure 2L).

After vascular rupture, blood enters the brain parenchyma and activates the complement system, leading to the rapid breakdown of RBCs and the subsequent release of large amounts of hemoglobin (Hb).^[^ ^37^ ^]^ To enhance the targeted delivery of the SDz nanostructures to the ICH site, a Hb aptamer was selected for the lesion site.^[^ ^38^ ^]^ Various nanostructures were co‐incubated with a Hb solution to mimic the ICH environment. After co‐incubation for 2 h, the nanostructures were centrifuged, and the supernatant was analyzed using ultraviolet/visible spectroscopy (UV–vis) (Figure S5, Supporting Information). Surprisingly, during the assessment of Hb trapping efficiency, it was observed that the different nanostructures did not significantly differ in their ability to recognize and capture Hb, whether or not the Hb aptamer was present (Figure S6, Supporting Information). Therefore, it was hypothesized that the DNAzyme itself might have the capacity to recognize and capture Hb. To further investigate the ability of the DNAzyme to recognize Hb, a magnetic bead experiment was conducted (Figure 2M). The dissociation constants (Kd) were determined through non‐linear regression fitting of the curves. As illustrated in Figure 2N, both the Hb aptamer and DNAzyme demonstrated high binding affinities, with Kd values of 19.02 and 37.64 nm, respectively. These findings indicate that the SIRPα DNAzyme sequence not only possesses the ability to cleave SIRPα but can also capture Hb, likely due to its unique secondary structure.

The stability of SDz in various in vitro environments was assessed. Initially, the effects of heating at 95 °C and the presence of different components, such as a culture medium containing 10% serum and nuclease cleavage, on SDz stability were examined. As shown in Figure S7 (Supporting Information), agarose gel electrophoresis indicated that SDz remained remarkably stable under these conditions. Furthermore, an evaluation of the stability of SDz after prolonged storage indicated no significant alterations in the particle size distribution, even after 7 days (Figure S8, Supporting Information). In contrast, traditional DNA self‐assembly materials have been reported to dissociate at low concentrations.^[^ ^39^ ^]^ To investigate the stability of SDz at low concentrations, the SDz‐containing system was diluted and measured after 1 h. As shown in Figure S9 (Supporting Information), SEM revealed no notable changes in the SDz surface after dilution. Furthermore, an in vitro hemolysis test demonstrated that SDz exhibited favorable blood compatibility and stability (Figure S10, Supporting Information).

### 2.2 Cellular Uptake and Phenotypic Regulation of SDz

**Figure 3:**
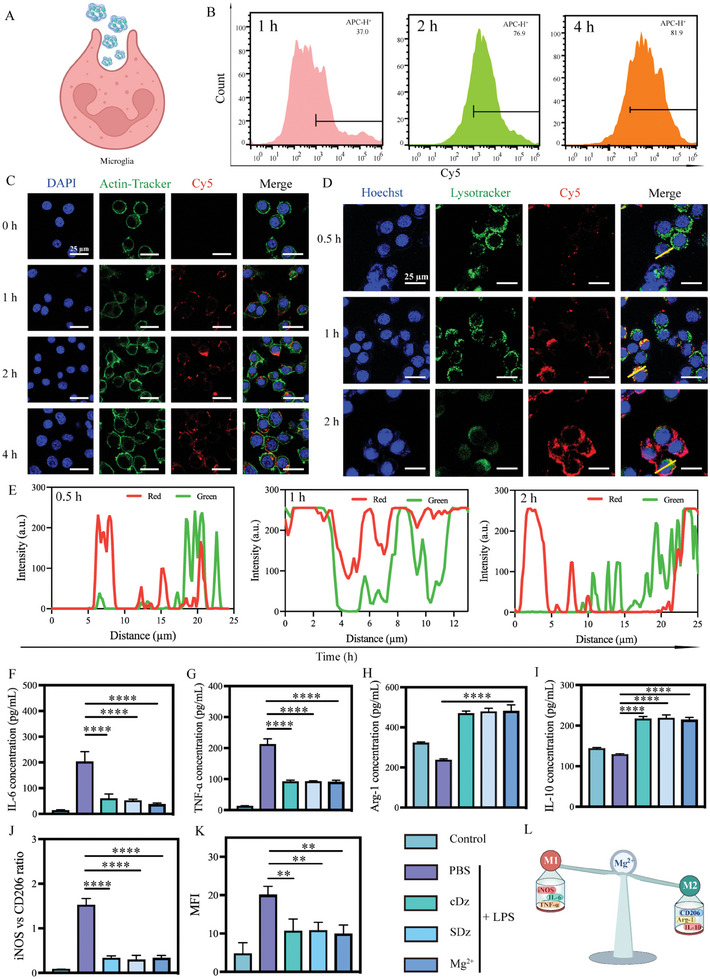
Cellular uptake and regulation of the microglial phenotype by SDz. A) Schematic diagram of SDz uptake by cells. B) Flow cytometry analysis of BV2 cells after incubation with SDz at different times. C) Representative CLSM images of Cy5‐labeled SDz in BV2 cells for different durations. Scale bar: 25 µm. Blue: DAPI; Green: actin‐tracker; Red: Cy5‐labeled SDz. D) Representative CLSM images of lysosomal Cy5‐labeled SDz in BV2 cells treated for different durations. Cell nuclei were stained with Hoechst. Scale bar: 25 µm. E) The intracellular distribution of Cy5‐SDz in the BV2 cells after different incubation times; the yellow line shows lysosomal labeling and Cy5‐SDz co‐localization, analyzed with Image J. Relative levels of F) IL‐6, G) TNF‐α, H) Arg‐1, and I) IL‐10 in BV2 cells. J) Semi‐quantitative analysis of iNOS and CD206 expression in BV2 cells after treatment with LPS (1 µg mL^−1^) and different nanostructures (n = 3). K) MFI of DCFH‐DA fluorescence in BV2 cells after treatment with nanostructures for 24 h for the determination of intracellular ROS (*n* = 3). L) Schematic diagram of microglial reprogramming and changes in factor expression. The results are shown as the mean ± standard deviation. **P < 0.01, ****P < 0.0001. Statistical analysis was performed using one‐way ANOVA followed by Tukey's HSD post hoc test (G, H, I, J, K, L). BV2, microglia; CLSM, confocal laser scanning microscopy; ROS, reactive oxygen species; LPS, Lipopolysaccharides; SDz, SIRPα‐DNAzyme nanostructures; cDz, complementary DNAzyme nanostructures; MFI, Mean fluorescence intensity.

Following the characterization of the nanostructure, the functioning of SDz at the cellular level was investigated. First, the potential toxicity of SDz was examined in various brain cell types, including cerebral vascular endothelial cells (bEnd.3 cells), microglia (BV2 cells), and hippocampal neuronal cells (HT‐22 cells). As shown in Figure S11 (Supporting Information), SDz exhibited no visible long‐term cytotoxicity toward any of these cell types. Additionally, the uptake of SDz by BV2 cells was assessed (**Figure** **3**A). BV2 cells were treated with Cy5‐SDz for varying durations, followed by analysis using flow cytometry (FCM) and confocal laser scanning microscopy (CLSM). The FCM results indicated a progressive increase in fluorescence intensity in BV2 cells with prolonged incubation (Figure 3B), a trend corroborated by the CLSM findings (Figures 3C and S12, Supporting Information). These results demonstrate the efficient internalization of SDz by BV2 cells.

In vitro verification confirmed that SDz could recognize and capture Hb (Figure 2M). Consequently, the impact of Hb captured by SDz on microglial activity was investigated. As shown in Figure S13 (Supporting Information), after capturing Hb, SDz was taken up by microglia without affecting their activity. Live/dead cell assays further confirmed that Hb captured by SDz did not significantly influence cell survival (Figure S14, Supporting Information). To further investigate the endocytic process of SDz and its potential to escape lysosomes, lysosomes in BV2 cells were labeled with Lysotracker to observe the dynamic changes associated with endocytosis. As shown in Figure 3D,E, the fluorescence of Lysotracker (green) and Cy5‐labeled SDz (red) co‐localized after 1 h of treatment, indicating that SDz was primarily distributed in lysosomes. However, after 2 h of treatment, the red SDz signal significantly increased and exhibited reduced co‐localization with the green signal, indicating that SDz escaped from the lysosomes and entered the cytoplasm.

After ICH, microglia undergo activation, leading to significant changes in both morphology and functionality.^[^ ^40^ ^]^ Activated microglia are now classified into two phenotypes: M1, which is pro‐inflammatory, and M2, which is anti‐inflammatory.^[^ ^41^ ^]^ M1 microglia secrete inflammatory factors, including interleukin‐6 (IL‐6) and TNF‐α, which can induce neuronal apoptosis, increase blood‐brain barrier permeability, and facilitate the infiltration of peripheral immune cells,^[^ ^42^ ^]^ thereby exacerbating the formation of the immune storm. In contrast, M2 microglia express a range of anti‐inflammatory factors, such as interleukin‐10 (IL‐10) and arginase‐1 (Arg‐1), which protect nerve cells and promote the recovery of neural function.^[^ ^43^ ^]^ The timely regulation of M1 microglia to M2 microglia at the ICH site is anticipated to enhance recovery from ICH.^[^ ^44^ ^]^ Therefore, the impact of SDz on the microglial phenotype was investigated. The regulatory effect of SDz on microglia was assessed by examining the expression of inflammatory factors (IL‐6, TNF‐α) and anti‐inflammatory factors (Arg‐1, IL‐10). Initially, BV2 cells were incubated with 1 µg mL^−1^ lipopolysaccharide (LPS) for 24 h, followed by the addition of nanostructures along with the equivalent Mg^2+^ content determined from the ICP‐MS results at pH 7.4 (Figure 2G). After an additional 24 h, cytokine levels were measured in the culture supernatants. The concentrations of IL‐6 and TNF‐α induced by LPS stimulation significantly decreased after incubating the cells with the nanostructures and Mg^2+^ (Figure 3F,G). In contrast, the concurrent upregulation of Arg‐1 and IL‐10 indicated a marked improvement in the inflammatory environment induced by LPS stimulation (Figure 3H,I). Furthermore, immunofluorescence was used to observe phenotypic changes in microglia, measuring the levels of inducible nitric oxide synthase (iNOS) and CD206 (markers for M1 and M2 phenotypes) in BV2 cells. As illustrated in Figures 3J and S15 and S16 (Supporting Information), the expression of iNOS significantly increased after LPS treatment in the PBS‐treated control group, indicating polarization toward the M1 microglial phenotype. In contrast, the expression of CD206 increased while that of iNOS decreased in the CDz, SDz, and Mg^2+^ groups. Additionally, dichlorodihydrofluorescein diacetate (DCFH‐DA) staining demonstrated that Mg^2+^ could reduce the levels of reactive oxygen species (ROS) (Figure 3K). These results suggest that Mg^2+^ may reverse M1 polarization while promoting M2 polarization (Figure 3L).

### 2.3 Mechanism of Polarization Regulation in Microglia

**Figure 4:**
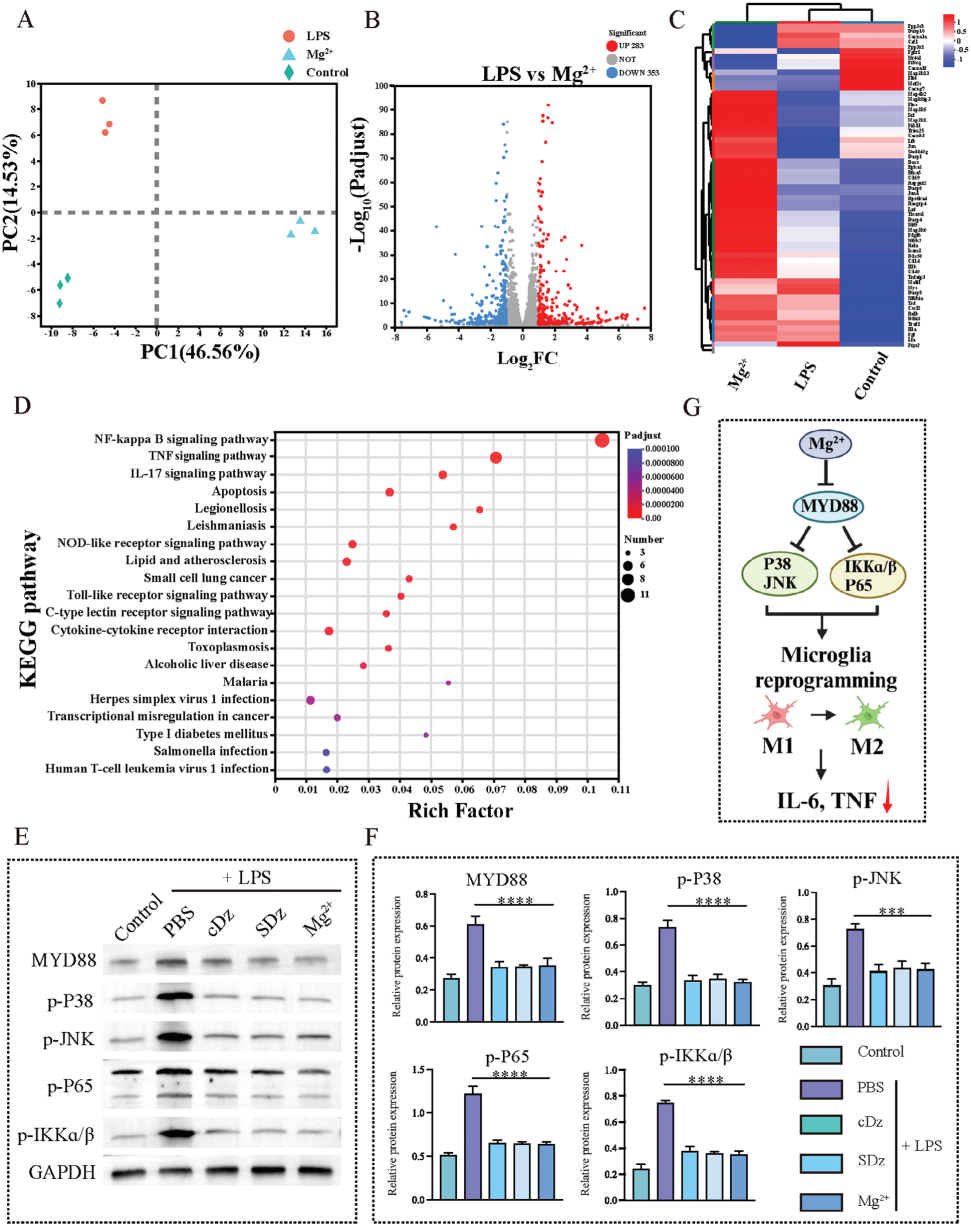
Mechanism of microglial polarization. A) PCA diagram showing the first principal component on the ordinate and the second principal component on the abscissa. B) Volcano plot of differentially expressed genes (DEGs) between the LPS‐ and Mg^2+^‐treated groups. Up‐regulated genes are represented by blue dots and down‐regulated genes by red dots. C) Volcano plot showing DEGs between Mg^2+^‐treated and LPS‐treated groups. D) KEGG pathways with significant enrichment in DEGs between the Mg^2+^ and LPS groups (*n* = 3). E) WB analysis of MyD88, p‐P38, p‐JNK, p‐P65, and p‐IKKɑ/β expression in BV2 cells after treatment with LPS (1 µg mL^−1^) and various treatments for 24 h (*n* = 3). F) Semi‐quantitative analysis of protein expression in BV2 cells after treatment with LPS and different nanostructures. G) Schematic illustrating the mechanism underlying the intracellular effects of Mg^2+^. The results are presented as the mean ± standard deviation. ***P < 0.001, ****P < 0.0001. Statistical analysis was performed using one‐way ANOVA followed by Tukey's HSD post hoc test (G, F). KEGG, Kyoto Encyclopedia of Genes and Genomes; LPS, Lipopolysaccharides; SDz, SIRPα‐DNAzyme nanostructures; cDz, complementary DNAzyme nanostructures.

To further investigate the mechanism by which Mg^2+^ modulated microglial polarization, RNA sequencing was performed on BV2 cells from the different treatment groups, with untreated BV2 cells serving as the control. Principal component analysis (PCA) revealed clustering of data within the same treatment groups, while significant differences were observed among the various groups, indicating the quality of the RNA sequencing results (**Figure** **4**A). Volcano plots revealed differentially expressed genes (DEGs) among the various treatment groups (Figure 4B). This analysis provided insights into the molecular mechanisms underlying the modulation of the inflammatory response mediated by Mg^2+^ in microglia. Specifically, the volcano plots indicated that 283 of the 636 DEGs were upregulated in Mg^2+^‐treated BV2 cells, while 353 were downregulated. Meanwhile, the heatmap revealed DEGs in response to LPS stimulation alone compared to LPS stimulation with Mg^2+^ treatment. Hierarchical clustering identified 63 representative DEGs (Figure 4C). The Kyoto Encyclopedia of Genes and Genomes (KEGG) pathway analysis (Figure 4D) and Gene Ontology (GO) analysis (Figure S17, Supporting Information) of the DEGs revealed an enrichment of genes involved in signal transduction and cytokine‐cytokine receptor interactions, including pathways related to TNF signaling, inflammation, Toll‐like receptor signaling, and NF‐κB signaling. Given the regulation of IL‐6 and TNF‐α by Mg^2+^, it was speculated that Mg^2+^ may regulate the microglial phenotype through inflammation‐related pathways. Next, the expression of relevant proteins in cells was evaluated by Western blotting (WB). The results indicated that, compared to the LPS group, there was a downregulation in the expression of MyD88, p‐IKKα/β, p‐P38, p‐P65, and p‐JNK in the cDz, SDz, and Mg^2+^ groups pretreated with LPS (Figure 4E,F).

LPS activated the MyD88/MAPK/NF‐κB signaling pathway, promoting the polarization of microglia to the M1 phenotype. In comparison to the LPS‐treated positive control group, treatment with Mg^2+^ significantly reduced the expression of these proteins. The activation of the transcription factor NF‐κB leads to its translocation from the cytoplasm to the nucleus, where it promotes the transcription of target genes, including those encoding pro‐inflammatory cytokines. The WB results further confirmed that Mg^2+^ effectively inhibited LPS‐induced phosphorylation of P65. Furthermore, LPS‐induced phosphorylation of P38/JNK was reduced following Mg^2+^ treatment, thereby inhibiting MAPK signaling and enhancing cell survival. Together, these data suggest that Mg^2+^ blocks the MyD88/MAPK/NF‐κB signaling pathway, thereby reducing inflammation through decreased expression of the pro‐inflammatory cytokines IL‐6 and TNF‐α (Figure 4G).

### 2.4 SDz‐Induced SIRPα Reduction and Enhanced Erythrophagocytosis in BV2 Cells

**Figure 5:**
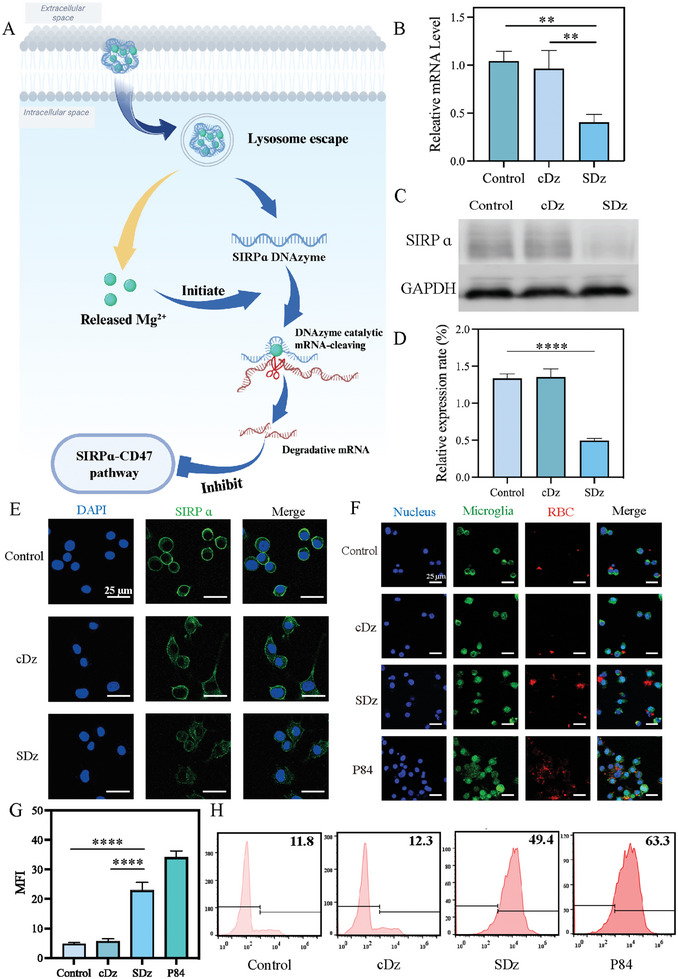
SDz‐induced reduction in SIRPα levels and enhanced RBC phagocytosis in BV2 cells. A) Schematic illustration of reduced expression by SDz in BV2 cells. B) qRT‐PCR measurement of SIRPα levels in BV2 cells after 24 h treatment with cDz or SDz (*n* = 3). WB C), semi‐quantitative analysis D), and immunofluorescence analysis E) of SIRPα protein in BV2 cells after treatment with different nanostructures (50 nm) for 24 h (*n* = 3). Scale bar: 25 µm. Blue: DAPI; Green: SIRPα. F) Representative fluorescence images showing phagocytosis of DiI‐labeled RBCs by microglia treated with cDz, SDz, or P84. The P84 monoclonal antibody specifically targets Signal Regulatory Protein α (SIRPα), and enhances the phagocytosis of red blood cells (RBCs) by microglia by blocking the interaction between SIRPα and CD47. Scale bar: 25 µm. Blue: nucleus; green: microglia (Iba‐1 marked); red: RBC. G) Semi‐quantification of immunofluorescence of DiI‐labeled RBCs phagocytized by microglia after 24 h treatment with different nanostructures (*n* = 3). H) Flow cytometry analysis of RBCs phagocytosis by BV2 cells treated with different nanostructures for 24 h. RBCs were stained with DiI. The results are shown as the mean ± standard deviation. **P < 0.01, ****P < 0.0001. Statistical analysis was performed using one‐way ANOVA followed by Tukey's HSD post hoc test (B, D, G). SIRPα, Signal‐Regulatory Protein α; SDz, SIRPα‐DNAzyme nanostructures; cDz, complementary DNAzyme nanostructures; PCR, polymerase chain reaction. RBC, red blood cell; Iba‐1, ionized calcium‐binding adaptor molecule‐1.

After ICH, RBCs, as the primary component of blood, infiltrate the brain parenchyma, inducing secondary brain injury and contributing to dysfunction and poor prognosis.^[^ ^45^ ^]^ As brain macrophages, microglia are rapidly activated and recruited to areas around the hematoma.^[^ ^46^ ^]^ However, CD47 on the surface of RBCs interacts with its ligand SIRPα expressed on microglia, providing a “self” signal that allows RBCs to evade recognition and phagocytosis by these cells.^[^ ^47^ ^]^ Therefore, blocking the CD47‐SIRPα interaction could inhibit the “self” signal, enhancing the phagocytosis of RBCs by microglia, thereby accelerating hematoma clearance and reducing damage to nerve cells.^[^ ^48^ ^]^ As illustrated in **Figure** **5**A, SDz released the SIRPα DNAzyme and Mg^2+^ in the acidic environment of the lysosome. Besides regulating the microglial phenotype, Mg^2^⁺ also served as a cofactor to activate the SIRPα DNAzyme, effectively silencing SIRPα and ultimately blocking the CD47‐SIRPα interaction.

To verify this, the cleavage of SIRPα mRNA was evaluated in SDz‐treated BV2 cells using quantitative real‐time PCR (qRT‐PCR). As shown in Figure 5B, there were significant reductions in SIRPα mRNA expression in the SDz group, with minimal changes in the other groups, demonstrating effective silencing of SIRPα. This was further confirmed by WB analysis, which showed a marked decrease in SIRPα protein levels in SDz‐treated BV2 cells (Figure 5C,D). The downregulation of SIRPα protein expression following SDz treatment was further confirmed by immunofluorescence. As shown in Figures 5E and S18 (Supporting Information), the green fluorescence signal in BV2 cells treated with SDz was significantly reduced compared to the control group, indicating effective inhibition of SIRPα protein expression. As illustrated in Figure 5F,G, CLSM observations demonstrated that SDz significantly enhanced the phagocytosis of RBCs by microglia. BV2 cells were pretreated with SDz or other control formulations for 24 h. Subsequently, DiI‐labeled RBCs and microglia were co‐cultured at a 5:1 ratio for 6 h, and the proportion of positive microglia (DiI^+^) in the microglial population was assessed, indicating the percentage of phagocytic microglia. As shown in Figure 5H, SDz treatment increased the percentage of phagocytic microglia to 49.4%, reflecting a 4.02‐fold increase compared to the cDz group. Moreover, by measuring the expression levels of iNOS and CD206, we demonstrated that the the increased phagocytosis of red blood cells by microglia under the treatment of SDz did not reverse the M2 microglia phenotype (Figure S19, Supporting Information).

### 2.5 Selective Targeting of Lesion Sites in Collagenase‐Induced ICH Models and the Therapeutic Effects of SDz

**Figure 6:**
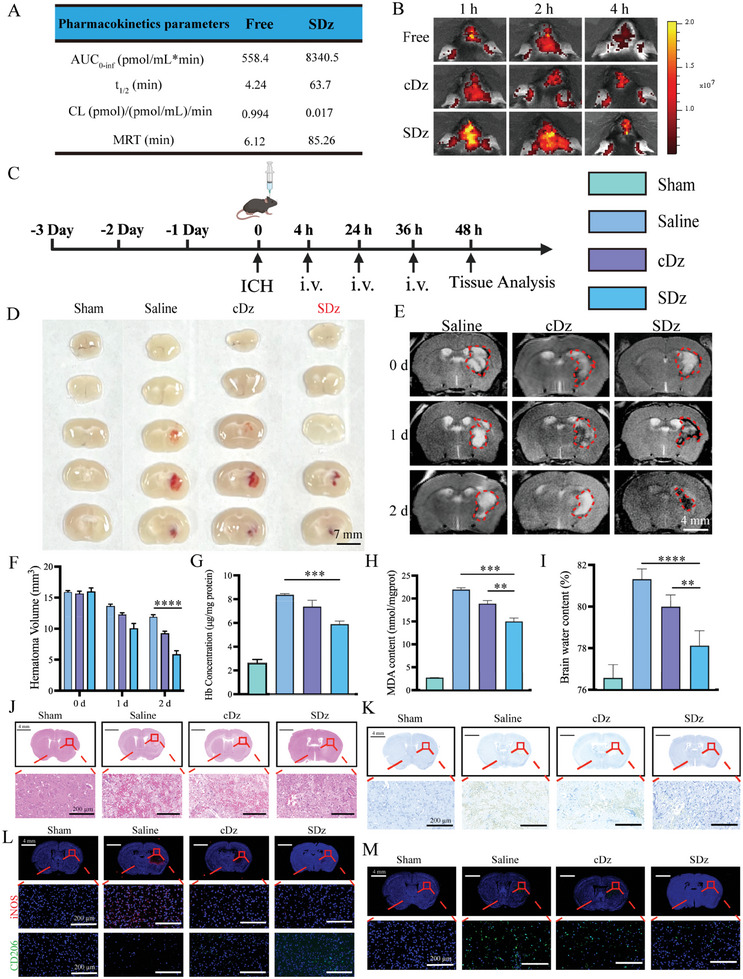
Preferential targeting of SDz to ICH regions and therapeutic efficacy of SDz in the collagenase‐induced mouse model of ICH. A) In vivo pharmacokinetic parameters of free DNAzyme and SDz (equivalent to 1 mg k^−1^g Cy5) after intravenous (i.v.) administration. B) In vivo distribution of free DNAzyme, cDz, and SDz in the collagenase‐induced ICH mouse model (*n* = 5). C) Schematic illustration of the design of the animal experiment. D) Representative images of brain sections from mice with ICH treated with saline, cDz, or SDz. E) Bleeding volumes were evaluated at different time points by magnetic resonance T2‐weighted imaging. F) Semi‐quantitative analysis of T2 lesion volume in the ipsilateral hemisphere. G) Hb concentrations in the brains of mice after different treatments, measured using Drabkin's reagent (*n* = 5). H) MDA contents in the brains of mice after different treatments (*n* = 5). I) Brain water contents of the mouse brains after different treatments, measured by the dry/wet weight method (*n* = 5). J–M) Representative images of (J) H&E (scale bar: 4 mm [the entire image] or 200 µm [the enlarged image]), (K) Nissl, (L) iNOS and CD206 staining, and M) TUNEL staining of brain sections from mice after different treatments (scale bar: 4 mm [the entire image] or 200 µm [the enlarged image]). The results are shown as the mean ± standard deviation. **P < 0.01, ***P < 0.001, ****P < 0.0001. Statistical analysis was performed using one‐way ANOVA followed by Tukey's HSD post hoc test (F, G, H, I). SDz, SIRPα‐DNAzyme nanostructures; cDz, complementary DNAzyme nanostructures; ICH, intracerebral hemorrhage; MDA, malondialdehyde; Hb, hemoglobin.

Following the encouraging in vitro results, an in‐depth study was conducted to investigate the therapeutic effects of SDz in vivo. Initially, free Cy5‐DNAzyme and an equivalent dose of Cy5‐labeled SDz were injected intravenously into mice. Blood samples were then collected at various time points (0, 5, 10, 15, 30, 60, 120, 240, and 480 min) to assess and quantify the fluorescence intensity in the plasma. As illustrated in **Figures** **6**A and S20 (Supporting Information), treatment with SDz significantly enhanced the area under the curve (AUC, 14.94‐fold), the half‐life (t_1/2_, 15.02‐fold), and the mean residence time (MRT, 13.93‐fold) compared to the free DNAzyme. More importantly, the blood clearance rate in the SDz group was 58.47 times lower than that in the free DNAzyme group, likely due to the high‐density DNA accumulation and the spherical structure of the SDz. To investigate whether SDz enhanced targeted delivery to the ICH, free Cy5‐DNAzyme, cDz, and SDz were injected into the mice. As shown in Figure 6B, the presence of SDz in the brains was examined using small‐animal live imaging. In vivo fluorescence imaging revealed prominent signals in the ICH lesions, indicating that SDz exhibited better brain targeting than free DNAzyme and cDz.

Next, the therapeutic effect of SDz on ICH in C57BL/6 mice was evaluated in vivo, following the treatment scheme outlined in Figure 6C. The hematoma size is a critical clinical predictor of prognosis in patients with ICH.^[^ ^49^ ^]^ As shown in Figure 6D, hematoma absorption was significantly greater in the SDz group compared to the cDz group. Additionally, the therapeutic effect of the drugs was assessed using T2‐weighted magnetic resonance imaging (MRI) scans. The hematoma volume in lesion areas exhibited high signal intensities in the right brain across the different treatment groups. The hematoma volumes following SDz treatment were significantly reduced compared to the saline group, decreasing from 11.90 to 5.84 mm^3^, which aligned with the findings from the brain sections (Figure 6E,F). Additionally, the Hb content in the brain was assessed using Drabkin's reagent. As anticipated, the trend in Hb content was consistent with the reduction in hematoma size (Figure 6G).

The entry of hematoma and its cleavage products into brain tissue causes damage to various types of brain cells, resulting in the infiltration of harmful substances and subsequent peroxidation of cellular lipids. To evaluate the protective effects of SDz, the malondialdehyde (MDA) levels in the brains of ICH mice were examined. The results revealed that MDA levels in the SDz group were significantly lower than those in the control group (Figure 6H). Intracranial hematomas can mechanically damage surrounding brain tissue, elevate intracranial pressure, and induce brain edema, potentially leading to brain herniation in severe cases. Reducing brain water content is a critical therapeutic goal in managing cerebral hemorrhage. To assess the effectiveness of treatments, the brain water content was measured across different treatment groups in the ICH mouse models. As shown in Figure 6I, the brain water content in the SDz‐treated group was significantly lower than that in the control group. Notably, mice treated with SDz exhibited only slight changes in body weight, whereas those treated with saline showed rapid weight loss (Figure S21, Supporting Information).

After 48 h of treatment, the mice were euthanized, and major organs were harvested for a series of analyses. The brain sections from mice in the different treatment groups were examined to further evaluate the efficacy of SDz in vivo. Initially, the morphological and structural characteristics of cytopathic changes at the ICH site were investigated using hematoxylin and eosin (H&E) staining. The results indicated significantly less brain damage in the SDz‐treated group compared to the control group (Figure 6J).

The ability of SDz to protect nerve cells in the brain is a crucial indicator in the treatment of ICH. Nissl staining revealed that mice treated with SDz exhibited the highest number of neurons (Figure 6K). To further investigate the effect of SDz on the microglial phenotype, the expression of various microglial phenotypic markers in the different treatment groups was assessed using immunofluorescence. The M1 marker (iNOS) and M2 marker (CD206) were selected for expression analysis (Figures 6L and S22, Supporting Information). The results indicated that SDz induced a shift in the microglial phenotype from M1 to M2. To evaluate brain cell apoptosis, the effect of SDz on cell activity was assessed using transferase‐mediated deoxyuridine triphosphate nick‐end labeling (TUNEL) staining, which confirmed the potent therapeutic efficacy of SDz (Figure 6M).

### 2.6 Improvement of Behavioral Function in ICH Mice

Neuronal damage can affect an animal's perception, movement, and other essential functions.^[^ ^50^ ^]^ Consequently, behavioral assessments in mouse models are essential for evaluating the efficacy of therapeutic interventions. These assessments objectively measure cognitive and motor functions, offering valuable insights into treatment outcomes. Continual monitoring of mouse behavioral changes not only tracks rehabilitation progress but also provides essential data for scientific analysis and optimization of experimental methods.

**Figure 7:**
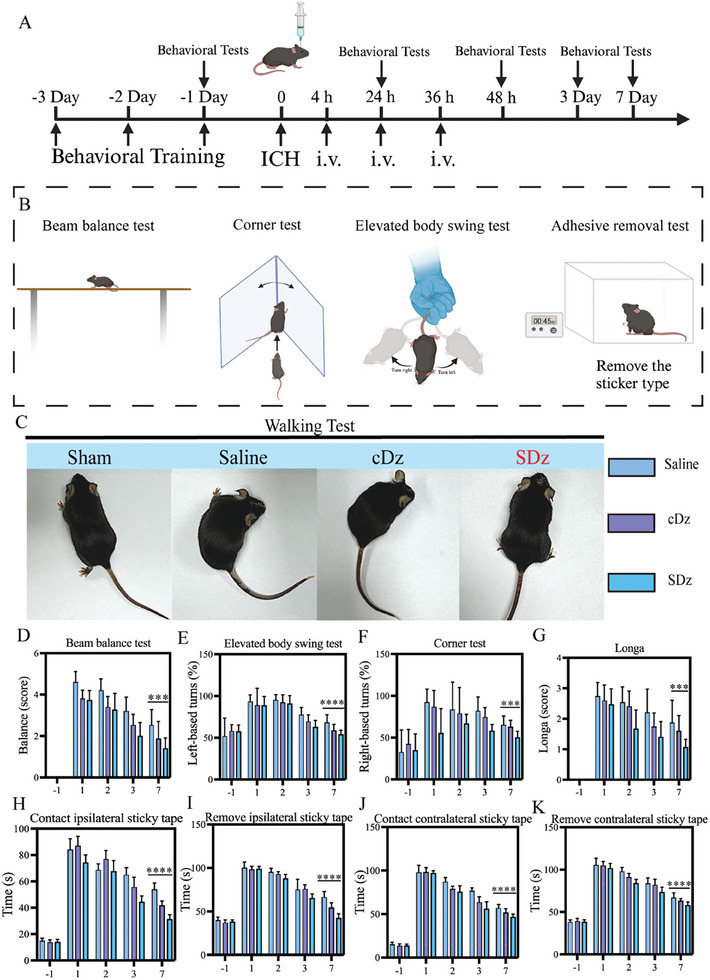
Neurological recovery in ICH mice models. A) Schematic representation of the schedule for behavioral training and testing. B) Schematic of behavioral testing, including the beam balance, elevated body swing, corner, and adhesive removal tests. C) Representative images of sham and ICH mice treated with saline, cDz, or SDz. D–K) Behavioral tests to verify the effects of SDz treatment included the D) beam balance test, E) elevated body swing test, F) corner test, G) Longa test, and (H‐K) sticky tape test (*n* = 15). Data are mean ± standard deviation. ***P < 0.001, ****P < 0.0001. Statistical analysis was performed using one‐way ANOVA followed by Tukey's HSD post hoc test (D‐K). SDz, SIRPα‐DNAzyme nanostructures; cDz, complementary DNAzyme nanostructures; ICH, intracerebral hemorrhage.

All animals were trained for at least 3 d before the experiment. The ICH model was then induced by intracerebral injection of collagenase, followed by SDz treatment. Behavioral tests were conducted to assess the therapeutic effects (**Figure** **7**A). In brief, functional rehabilitation in ICH mice was evaluated using modified neurological severity scores, which included neurological deficit tests, as well as beam balance, corner, and adhesive removal tests (Figure 7B–K). Mice treated with SDz demonstrated the ability to walk in a straight line and exhibited lower neurological severity scores compared to other treatment groups. Additionally, behavioral tests, including the beam balance, corner, elevated body swing, and adhesive removal tests, showed significant post‐operative recovery in the SDz‐treated mice. The behavioral experiments demonstrated that ICH significantly impaired various motor and perceptual functions in mice, while SDz treatment markedly improved neuromotor abilities. The comprehensive assessment of the mice confirmed the protective role of SDz against ICH, highlighting its excellent therapeutic efficacy.

A comprehensive assessment of the biosafety of SDz was conducted by staining pathological sections of major organs (heart, liver, spleen, lung, and kidney) with HE. The results, presented in Figure S23 (Supporting Information), indicated no significant morphological changes in the tissues following SDz treatment, demonstrating that SDz poses no significant toxicity to the major organs. In addition, analyses of hematology (Figure S24, Supporting Information) and liver and kidney function (Figure S25, Supporting Information) were conducted, clearly demonstrating that SDz is biocompatible and has negligible effects on the mice. In summary, these results indicate that SDz is a safe and effective treatment for mice with ICH.

## 3 Conclusion

In conclusion, this study introduces a novel nanoregulator designed to enhance hematoma resolution and suppress neuroinflammation following ICH. Treatment with SDz significantly accelerated hematoma absorption and reduced neurological inflammation, as evidenced by decreased secretion of pro‐inflammatory cytokines and increased production of anti‐inflammatory factors. Furthermore, the neurological deficits observed in mice with ICH were markedly alleviated. The implementation of a co‐delivery strategy, combined with the complex synergistic interactions within SDz, resulted in enhanced therapeutic efficacy against ICH without any apparent systemic side effects. The potential of SDz to simultaneously address both hematoma resolution and neuroinflammation underscores its significance as a promising therapeutic avenue for ICH, providing a novel and comprehensive approach to improving patient outcomes.

This straightforward yet highly effective nanoplatform offers substantial promise for introducing innovative perspectives into clinical and translational therapies for ICH. However, advancing SDz to clinical practice needs further in vivo and pre‐clinical studies to substantiate its therapeutic potential. Moreover, it is essential to ensure its safety and efficacy in human trials to ensure its feasibility and accessibility to patients. Additionally, addressing the cost and complexity of scaling nucleic acid medicine production is crucial for realizing clinical adoption.

## 4 Experimental Section

### Reagents

All DNA sequences were synthesized and purchased from Sangon Biotech Co., Ltd. (Shanghai, China). T4 DNA ligase and phi29 DNA polymerase were obtained from Thermo Fisher Scientific Co., Ltd. (Shanghai, China). Deoxy‐ribonucleoside triphosphates (dNTPs) were sourced from Takara Biomedical Technology Co., Ltd. (Beijing, China), while the DNA marker was acquired from Real‐Times Biotechnology Co., Ltd. (Beijing, China). Cell Counting Kit‐8 (CCK‐8), DiI, FITC‐Phalloidin (CA1620) and 4′,6‐Diamidino‐2′‐phenylindole (DAPI) were obtained from Solarbio Science & Technology Co., Ltd. (Beijing, China). The quantitative real‐time PCR primers were purchased from Shanghai Gemma Pharmaceutical Technology Co., Ltd. (Shanghai, China). MYD88, p‐P38, p‐JNK, p‐P65 and GADPH antibody were purchased from Proteintech (Wuhan, China), and p‐IKKα/β antibody was acquired from Abcam (Cambridge, UK). The SYBR Green qPCR kit was obtained from TransGen Biotech. Mouse tumor necrosis factor‐α (TNF‐α) and interleukin‐6 (IL‐6) enzyme‐linked immunosorbent assay (ELISA) kits were acquired from Elabscience Biotechnology Co., Ltd. (Wuhan, China) and Lianshuo Biotechnology Co., Ltd, respectively. The anti‐SIRPα (EPR16264) and anti‐iNOS (ab178945) antibodies were obtained from Abcam (Cambridge, UK), while the Alexa Fluor 488 anti‐mouse CD206 (MMR) antibody was purchased from Bioss Biotechnology Co., Ltd. Interleukin‐10 (IL‐10) enzyme‐linked immunosorbent assay (ELISA) kits were acquired from Shanghai Jianglai Biotechnology Co., Ltd. Human hemoglobin (H7379) and collagenase type IV were obtained from Sigma‐Aldrich (St. Louis, MO, USA). The Malondialdehyde (MDA) Detection Kit was purchased from Nanjing Jiancheng Bioengineering Institute (Nanjing, China). All other reagents and solvents were purchased from Thermo Fisher Scientific and used as received unless otherwise specified.

### Cell Culture and Animals

Mouse microglia (BV2 cells), mouse hippocampal neurons (HT‐22 cells), and mouse brain microvascular endothelial cells (bEnd.3) were obtained from iCell Bioscience Inc. (Shanghai, China). The cells were cultured in Dulbecco's Modified Eagle Medium (DMEM) (Solarbio Science & Technology Co., Ltd.) supplemented with 10% fetal bovine serum (FBS) (Nanjing BioChannel Biotechnology Co., Ltd., China) in a 5% carbon dioxide atmosphere at 37 °C.

Male C57BL/6 mice (8 weeks, 20–22 g) were obtained from Si Pei Fu Biotechnology Co. Ltd. (Beijing, China; license No. 110 324 231 106 272 823). The mice were housed in an environment maintained at 55% humidity and 25 °C with a 12‐h light/dark cycle. All animal experiments were conducted following the Life Sciences Ethical Review Committee of Zhengzhou University, with accreditation number SCXK (YU) 2023‐0004.

### Synthesis of SDz and cDz

SDz was synthesized using the Rolling Circle Amplification (RCA) reaction. The 5′‐phosphorylated DNAzyme template and primer (sequences listed in Table S1, Supporting Information) were added to the T4 DNA ligase buffer. Specifically, the DNAzyme template (10 µm) and primer (10 µm) were combined in T4 DNA ligase buffer (1×) and incubated at 95 °C for 5 min, then gradually cooled to 25 °C at a rate of 0.1 °C s. Following this, T4 DNA ligase (0.25 U µL^−1^) was added, and the ligation reaction was allowed to proceed at room temperature, resulting in the formation of the circular DNAzyme template. The ligation circle was then incubated with phi29 DNA polymerase (0.125 U µL^−1^) and dNTPs (1 mm) in phi29 DNA polymerase buffer (1×) at 37 °C for 3 h. The reaction was terminated by inactivating phi29 DNA polymerase at 95 °C for 5 min. To purify the mixed RCA products, the terminated reaction mixtures were centrifuged at 12000 rpm for 20 min and then resuspended in nuclease‐free water. The synthesis of cDz followed the same procedure as described above.

### Characterization of the SDz

The template, primer, circular template, and SDz were analyzed by 2% agarose gel electrophoresis at 120 V for 50 min. The morphology of the samples was evaluated using SEM (SU8020, Japan) and TEM (SU8020, Japan) after complete drying. The size distribution and zeta potential of SDz were measured using DLS (Malvern Instruments Ltd., UK). The elemental composition of SDz was analyzed by energy‐dispersive X‐ray spectroscopy (EDS) and mapping (FEI Tecnai G2, USA).

### The Stability of SDz In Vitro

FBS (10%) and DNase I (2 U mL^−1^) were added to the SDz (500 nM) solution and incubated at 37 °C for 1 h. Additionally, another SDz solution was incubated at 95 °C for 20 min. After incubation, DNA loading buffer was added achieve a final concentration of 1×, and the samples were analyzed on 2% agarose gels. DLS was employed to monitor changes in the particle size of SDz over 7 days to assess its stability. For SEM analysis, the formulation was diluted 100 times and allowed to stand for 1 h.

### In Vitro Mg^2+^ Release from SDz

SDz (500 nm) was dissolved in PBS (pH 7.4 and 5.5) for 24 h and centrifuged at 12000 rpm for 20 min. The supernatant was collected, and the magnesium ion content was determined by ICP‐MS. The morphology was analyzed using SEM.

### PAGE Analysis

The DNAzyme‐mediated cleavage reaction was detected using PAGE. Different concentrations of Mg^2+^ were added to a mixture of DNAzyme (100 µm) and SIRPα substrate (100 µm) for 12 h. The sample was then diluted with loading buffer and applied to a freshly prepared 20% polyacrylamide gel. After electrophoresis for 1 h in 1×TB buffer (40 mm Tris, 89 mm boric acid, pH 8.3) at a constant voltage of 100 V, the gel was stained for 20 min in diluted GelRedTM solution, followed by imaging.

### Fluorescence‐Resonance Energy Transfer (FRET)

First, 50 µL of a 100 µm DNAzyme solution was mixed with a 100 µm SIRPα substrate solution for 3 h. Then, 50 µL of MgSO_4_ was added to achieve a final Mg^2+^ concentration of 20 µm. Immediately afterward, 50 µL of a mixture containing 50 µm fluorescent chain and 50 µm quenching chain was added to the solution. Finally, the real‐time fluorescence intensity was monitored using a microplate reader (Ex: 650 nm, Em: 670 nm).

### DNAzyme and SDz Cleavage In Vitro

In vitro cleavage by the DNAzyme was evaluated by incubating 500 nm of DNAzyme and 500 nm of SIRPα substrate in Tris‐HCl buffer (pH 8.0) with varying MgSO_4_ concentrations (0, 1, and 10 mm) at 37 °C for 12 h. After incubation, cleavage efficiency was analyzed by PAGE. Various concentrations of SDz were incubated in PBS (pH 7.4 and 5.5) for 12 h. The pH of the solution was then adjusted to 7.4, after which 100 µm of SIRPα substrate was added. Following a 5‐h incubation, cleavage efficiency was verified by PAGE.

### Hemolysis Assay

Fresh whole blood was collected into heparin from the retro‐orbital sinuses of mice. The blood was washed twice with pre‐cooled PBS and centrifuged (4 °C, 720 g, 5 min) to obtain the hematocrit. Subsequently, 1 mL of red blood cell (RBC) suspension (containing 20 µL of hematocrit) was incubated with various nanostructures (10 nm) for 3 h. Triton X‐100 served as the positive control, while PBS acted as the negative control. Representative images were captured at 0, 1, 2, and 3 h. After centrifugation (4 °C, 720 g, 5 min), the supernatants were collected, and the hemolysis rate was measured at 570 nm.

### In Vitro Cytotoxicity Assay

CCK‐8 colorimetric assays were conducted to assess the effects of SDz on cell viability and toxicity. Briefly, the cells were seeded in 96‐well plates at a density of 1 × 10^4^ cells per well and cultured in 100 µL of fresh culture medium containing 10% FBS. Once the cells had adhered to the plate, the medium was replaced with fresh culture medium containing different concentrations of SDz (0, 10, 20, 50 nm) for 24 h. Following this, the CCK‐8 solution was added and incubated in the dark at 37 °C for 1 h. The absorbance at 450 nm was then measured using a microplate reader (Synergy H1, USA). Cell viability and toxicity at different concentrations of SDz were calculated using the following formula (the OD represents the absorbance value of each well):

(1) $$\mathrm{cell}\;\mathrm{viability}\;\;\left(\%\right)=\frac{OD_{\mathrm{SDz}}-OD_{\mathrm{blank}}}{OD_{\mathrm{control}}-OD_{\mathrm{blank}}}\;\;\times \;100\%$$

### Cellular Uptake and Lysosome Escape

The efficiency of SDz cellular uptake was analyzed by CLSM and FCM. BV2 cells were inoculated in 24‐well plates (3 × 10^5^ cells per well) for 24 h. After washing with PBS, the cells were incubated with SDz for 0, 1, 2, and 4 h. Cellular uptake was subsequently assessed using CLSM and FCM. Lysosome escape was assessed by CLSM. BV2 cells were incubated with 50 nm SDz for 0.5, 1, and 2 h. After washing twice with PBS, 50 nm Lyso‐Tracker Green was added, and the cells were cultured at 37 °C with 5% CO_2_ for 1 h. The nuclei were stained for 20 min. After rinsing twice with cold PBS, the cells were immediately evaluated using a laser confocal microscope.

### IL‐6, TNF‐α, Arg‐1, and IL‐10 ELISA

BV‐2 cells were cultured in 10 cm cell culture dishes and treated with LPS (1 µg mL^−1^) along with various nanostructures (50 nm) for 24 h. The supernatants from each group were collected, and the concentrations of IL‐6, TNF‐α, Arg‐1, and IL‐10 were measured using different mouse ELISA kits.

### Quantitative Real‐Time Polymerase Chain Reaction (qRT‐PCR)

BV‐2 cells were incubated with different nanostructures for 24 h, after which total RNA was extracted using TRIzol reagent (ComWin Biotech Co., Ltd., China) for qRT‐PCR analysis. Reverse transcription was performed using a one‐step transcriptional gDNA removal and cDNA synthesis SuperMix (Transgenic Biotechnology, China). qRT‐PCR was conducted with the TSINGKE TSE201 2×TSINGKE Master qPCR Mix (SYBR Green I) (Tsingke Biotechnology Co., Ltd., China).

### Investigation of Microglial Polarization

BV‐2 cells were inoculated into 24‐well plates at a density of 1 × 10^5^ cells per well and allowed to adhere. The cells were then treated with LPS (1 µg mL^−1^) in combination with cDz, SDz (50 nm), or Mg^2+^ (20 µm). After 24 h of incubation, the levels of iNOS and CD206 were assessed. For immunostaining, the cells were washed three times with PBS and fixed with 4% paraformaldehyde for 15 min. Subsequently, permeabilization was performed using Immunostaining Permeabilization Solution with Triton X‐100 (Beyotime Biotech Co., Ltd.) for 10 min, followed by blocking with QuickBlock Blocking Buffer for Immunol Staining (Beyotime Biotech Co., Ltd.) for 30 min. The anti‐iNOS (1:500) and anti‐CD206 (1:200) antibodies were added and incubated with the cells overnight at 4 °C. Following washes with PBS, donkey anti‐rabbit IgG‐Alexa Fluor 594 secondary antibody was applied to detect iNOS expression, while Alexa Fluor 488 secondary antibody was used for CD206 detection. Both secondary antibodies were incubated at room temperature, protected from light, for 1 h. After staining the nuclei of BV2 cells with DAPI for 10 min in the dark, the expression levels of iNOS and CD206 were observed using CLSM).

### Western Blotting

BV‐2 cells were grown in 10 cm cell culture dishes at the same density and cultured until reaching 90% confluence. The cells were then treated with cDz or SDz (50 nm) under various conditions for an additional 24 h. After treatment, the cells were collected, and total cellular proteins were lysed using radioimmunoprecipitation (RIPA) buffer supplemented with 1 mm phenylmethylsulfonyl fluoride (PMSF). The total protein concentration was quantified using a BCA protein assay kit (Solarbio Science & Technology Co., Ltd.). Subsequently, the proteins were separated by 10% SDS‐PAGE and transferred to polyvinylidene fluoride (PVDF) membranes. The membranes were then blocked with 5% skimmed milk for 1 h. The blots were then incubated with anti‐SIRPα (1:1000) and anti‐GAPDH (1:8000) antibodies at 4 °C overnight. Following this, the membranes were incubated with a secondary antibody conjugated with horseradish peroxidase at 37 °C for 2 h. The bands were visualized using BeyoECL Star (Beyotime Biotechnology Co., Ltd.) and analyzed with Image Lab software (Bio‐Rad).

### In Vitro RBC Phagocytosis by Microglia

Mouse microglia were inoculated in 24‐well plates overnight. RBCs were separated from mouse whole blood by centrifugation. Different nanostructures labeled with Cy5 were added and incubated for 24 h. Following this, the RBCs were labeled with the fluorescent dye DiI (Beyotime Biotechnology Co., Ltd.) and subsequently added to the microglia at a ratio of 5:1 for an additional 6 h. For immunostaining, the cells were fixed with 4% paraformaldehyde for 15 min, followed by permeabilization using Immunostaining Permeabilization Solution with Triton X‐100 (Beyotime Biotech Co., Ltd.) for 10 min. Subsequently, the cells were blocked with QuickBlock Blocking Buffer for Immunol Staining (Beyotime Biotech Co., Ltd.) for 30 min. The cells were then co‐cultured with the anti‐Iba‐1 antibody (1:200) at 4 °C overnight, followed by incubation with donkey anti‐rabbit IgG‐Alexa Fluor 488 (1:200) at room temperature for 1 h. Afterward, the cells were stained with DAPI in the dark for 10 min and observed using CLSM.

### Collagenase‐Induced Mouse Model of Intracerebral Hemorrhage (ICH)

Collagenase was injected into the right striatum of the mice to establish the ICH mouse model. Male C57BL/6 mice (8 weeks, 20–22 g) were anesthetized with isoflurane and fixed in a stereotactic device for surgery. A hand‐held small skull drill was used to drill a hole of 0.8 mm in the right striatum. A miniature syringe with a glass microelectrode on the needle was placed into the mouse's brain through the hole. The positions of the insertion point were 0.5 mm in front of the leading edge, 2.0 mm outside the midpoint, and 3.2 mm deep in the craniofacial region. For the collagenase injection, 0.0375 U of collagenase (type IV, Sigma‐Aldrich) was administered into the right striatum at a rate of 0.05 µL min^−1^. The syringe was held in place for 10 min to prevent collagenase reflux. The mice in the sham surgery group were anesthetized with isoflurane, drilled with the skull drill, and then injected with saline using a micro‐syringe. Body temperature was maintained with a heating blanket throughout the procedure. During surgery, the mice were placed in a constant temperature incubator until awake. Surgically treated animals were randomly assigned to the saline, cDz, and SDz groups. Mice in these groups received drug treatment via tail vein injection at a dose of 5 nmol kg^−1^ at three time points: 4, 24, and 36 h post‐collagenase injection.

### In Vivo Pharmacokinetic Assay

To evaluate the pharmacokinetics of SDz, a single dose of Cy5‐labeled SDz or DNAzyme (equivalent to 1 mg kg^−1^ Cy5) in 200 µL PBS was injected into the mice via the tail vein (*n* = 3). At designated time points after injection, blood was extracted from the retro‐orbital sinus and mixed with 5 µL of heparin solution. Following centrifugation at 3000 g for 5 min, the supernatant was collected, and the fluorescence intensity of Cy5 was measured. Pharmacokinetic parameters were calculated using a single‐chamber model.

### In Vivo Imaging

ICH model mice were injected with cDz or SDz labeled with the same concentration of Cy5 (equivalent to 1 mg kg^−1^ Cy5). The brain‐targeting effect of SDz was studied using a small‐animal imager.

### Brain Tissue Collection, Immunohistochemistry, and Immunofluorescence Staining

ICH model mice treated with different nanostructures were anesthetized with isoflurane and euthanized 48 h after the operation. The brains were harvested immediately for subsequent analysis. The protective effect of SDz on ICH in mice was evaluated using H&E and Nissl staining to assess tissue morphology, along with TUNEL staining to detect apoptosis. Additionally, brain sections were stained with anti‐iNOS and anti‐CD206 antibodies.

### Magnetic Resonance Imaging (MRI)

MRI of the mouse brains was conducted in vivo using a 7‐T MRI system (Biospec 70/30; Bruker, USA). Mice were anesthetized with isoflurane gas during the examination. T2‐weighted imaging was performed utilizing a fast‐spin echo sequence, and the images were captured as 256 × 256‐pixel pictures. The hematoma volumes were subsequently analyzed using ImageJ. All measurements were repeated five times.

### Haemoglobin (Hb) Content

Mouse brains were perfused with pre‐cooled PBS and the Hb contents were measured to eliminate resident intravascular Hb. Following this, the ipsilateral hemisphere was homogenized in 500 µL of water at 4 °C. The homogenate was centrifuged at 4 °C and 12000 g for 30 min, after which the supernatant was combined with Drabkin's reagent in a 1:4 (v/v) ratio and incubated at 37 °C for 15 min. The absorbance at 540 nm was measured to determine the Hb content, using a standard curve generated by adding varying amounts of Hb to 500 µL of water and measuring the absorbance at 540 nm with Drabkin's reagent. Furthermore, the total protein content was determined using the BCA protein assay kit. The formula for calculating the Hb content was as follows:

(2) $$\begin{array}{c} \mathrm{Hb}\;\mathrm{content}\;\left(\mu g/\mathrm{mg}\;\mathrm{protein}\right)=\frac{\mathrm{Hb}\;\mathrm{concentration}\;(\mu g/\mathrm{mL})}{\mathrm{total}\;\mathrm{protein}\;\mathrm{concentration}\;(\mathrm{mg}/\mathrm{mL})} \end{array}$$

### Malondialdehyde (MDA) Measurement

Samples of brain tissue were homogenized in pre‐cooled Tris buffer (1:4 [w/v], 50 mm, pH 7.4) at 4 °C and centrifuged (4 °C, 10 000 *g*, 10 min). The supernatant was collected and the MDA content was quantitatively measured by the MDA assay kit. The protein content in the supernatant was also assessed using the BCA protein assay kit. The MDA content was expressed as nmol MDA/mg total protein.

### Brain Edema

The water content of the brain was measured using the dry/wet weight method. The ipsilateral hemisphere of the brain, excluding the olfactory bulb and cerebellum, was isolated, and the weight was recorded before and after drying at 80 °C for 72 h. The formula used to calculate the water content of the brain was as follows:

(3) $$\mathrm{brain}\;\mathrm{water}\;\mathrm{content}\;\;\left(\%\right)=\frac{\mathrm{wet}\;\mathrm{weight}-\mathrm{dry}\;\mathrm{weight}}{\mathrm{wet}\;\mathrm{weight}}\;\;\times \;100\%$$

### Behavioral Tests

Three researchers conducted behavioral tests on the mice while remaining blinded to the group assignments. Various assessments were performed to evaluate balance, motor function, and sensory function, utilizing the Longa test, sticky‐tape test, corner‐turning test, beam balance test, and elevated body swing test. These tests were conducted at different time points both before and after the collagenase injection.

### Longa Test

Mice were evaluated for motor and cognitive function using a four‐point scale:

4 = inability to walk spontaneously, loss of consciousness
3 = circling to the right only
2 = tendency to circle to the right
1 = failure to extend the right forelimb
0 = no deficit

### Elevated Body Swing Test

The mouse was suspended by the tail 10 cm above the platform surface, and each time the mouse turned its head to either side of the vertical axis was recorded as one swing. The percentage of swing preference was then calculated.

### Corner‐Turning Test

Mice were placed in a corner with two flat plates at a 30° angle and a small seam at the junction to attract the mouse into the corner. Healthy mice turned either left or right, while mice with ICH tended to turn right. The number of right turns was recorded over 10 tests, and the percentage of right turns was subsequently calculated.

### Sticky‐Tape Test

The left or right forelimb of the mice was taped, and healthy mice typically attempted to remove the tape using their mouths or the contralateral limb. The time taken for each mouse to successfully remove the tape was recorded.

### Beam‐Balance Test

A square beam (1 cm wide, 20 cm high, 100 cm long) was utilized to assess motor coordination and integration in mice. The scoring was based on a four‐point scale as follows:

4 = unable to remain on the balance beam
3 = unable to walk but could stay on the balance beam
2 = attempted to cross the balance beam but failed
1 = able to cross the balance beam but hind legs tended to slip off
0 = passed over the balance beam quickly.

Mice were trained to navigate the balance beam for three days before the model was established.

### Blood Biochemistry Measurement

The mice were treated with PBS or various treatment groups through in situ injection at a dosage of 5 nmol kg^−1^. Following treatment, blood samples and major organs (heart, liver, spleen, lungs, and kidneys) were collected for analysis. Blood samples were utilized to determine biochemical and hematological parameters, while histological analyses were conducted to assess the biosafety of the different nanostructures.

### Statistical Analysis

All data were presented as the mean ± standard deviation from a minimum of three independent experiments. Pre‐processing and statistical analysis relevant information was indicated in the experimental section or in the figure legends. Data analysis was conducted using GraphPad Prism (8.01). One‐way analysis of variance (ANOVA) followed by Tukey's honest significant difference (HSD) post hoc test was employed for multiple‐group comparisons, while unpaired Student's t‐tests were used for two‐group comparisons. Differences between the experimental and control groups were considered statistically significant at *P* < 0.05; *P < 0.05, **P < 0.01, ***P < 0.001, ****P < 0.0001.

### Ethical Approval Statement

All animal experiments were conducted following Zhengzhou University's Guide for the Care and Use of Laboratory Animals. The animal license number was No. 110 324 231 106 272 823, and the study received ethical approval under number SYXK‐20230004. Before the experiment, the animals were acclimated under controlled conditions at 25 °C, with 70 ± 5% relative humidity, and a 12‐h light/dark cycle for 7 days. In this study, the trauma experienced by the experimental mice was primarily due to pain resulting from ICH and tail vein injections. Isoflurane was administered for anesthesia during the modeling process. To minimize the pain and distress of the mice, a clean and comfortable living environment was provided, along with adequate food and water. The authors have adhered to the ARRIVE guidelines throughout the study.

## Conflict of Interest

The authors declare no conflict of interest.

## Author Contributions

J.S. and W.Y. conceived and designed the experiments. C.C. and W.Y. performed the experiments with assistance from Y.Y. and Y.Z. W.Y. wrote the manuscript. J.S. revised the manuscript. J.L., J.S., and A.C. supervised the entire project.

## Supporting information



Supporting Information

## Data Availability

The data that support the findings of this study are available in the supplementary material of this article.
