# Supplementary material for: Bioactive Self‐Assembled Nanoregulator Enhances Hematoma Resolution and Inhibits Neuroinflammation in the Treatment of Intracerebral Hemorrhage
Source: Adv Sci (Weinh). 2024 Nov 8;12(1):2408647. doi: 10.1002/advs.202408647 (PMC11714160; doi:10.1002/advs.202408647)
Supplement: Supplementary file 1 — Supporting Information [file ADVS-12-2408647-s001.docx]

Supporting Information

Bioactive self-assembled nanoregulator enhances hematoma resolution and inhibits neuroinflammation in the treatment of intracerebral hemorrhage

*Wenyan Yu, Chengyuan Che, Yi Yang, Yuzhen Zhao, Junjie Liu^*^, Aibing Chen^*^, Jinjin Shi^*^*


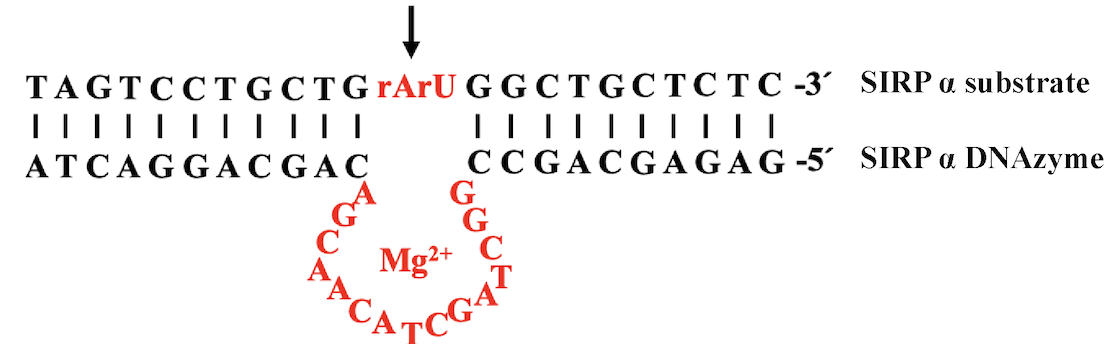


Figure S1. The secondary structure of the DNAzyme-SIRPα substrate complex. The arrow indicates the cleavage site on SIRPα. rA represents ribonucleic adenine, and rU represents ribonucleic uracil, which is more liable to cleavage than deoxyribonucleotides. In the SIRPα DNAzyme sequence, the red segment represents the catalytic domain, which is specifically activated by Mg^2+^ ions. The black segment denotes the two substrate-recognition sequences. SIRPα stands for Signal-Regulatory Protein α.


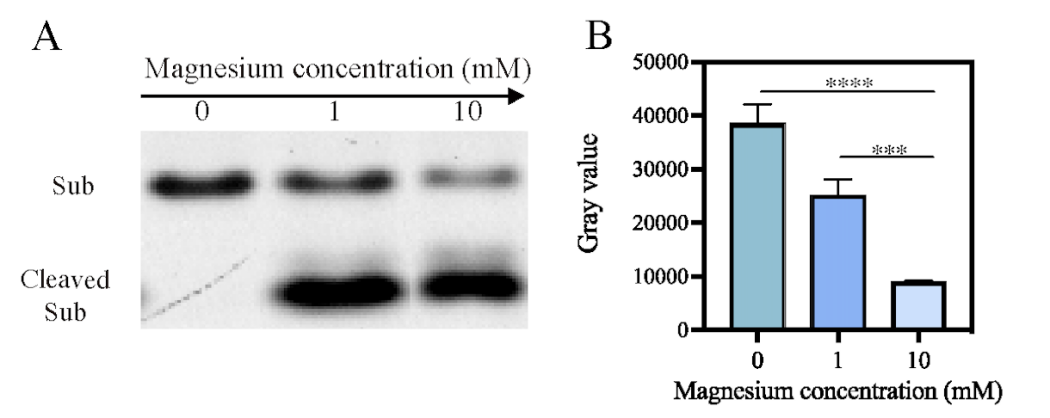


Figure S2. Denaturing PAGE gel (A) and semi-quantitative analysis (B) of SIRPα cleavage in the presence of different concentrations of Mg^2+^ (n=3). Data are expressed as mean ± standard deviation. ****P* < 0.001, *****P* < 0.0001. Statistical significance was calculated using one-way analysis of variance (ANOVA) followed by the post hoc Tukey test (B). PAGE, polyacrylamide gel electrophoresis.


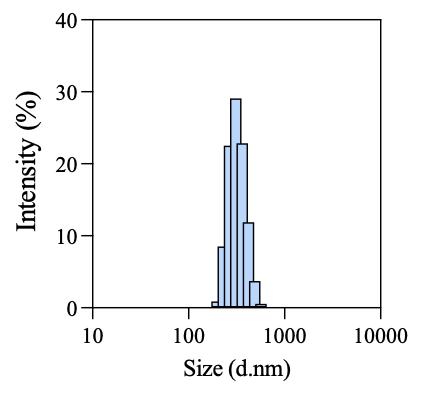


Figure S3. The size distribution of SDz, assessed using DLS. DLS, Dynamic light scattering.


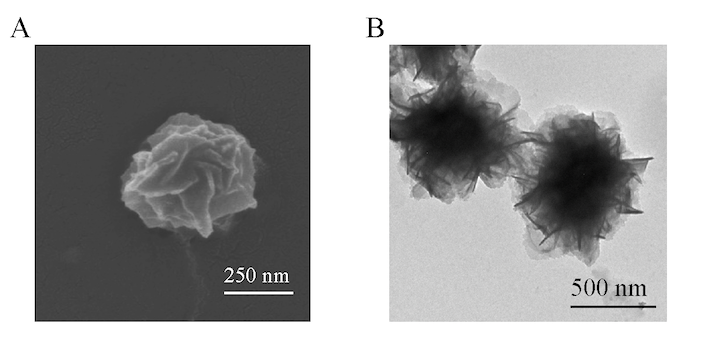


Figure S4. (A) Representative SEM image of cDz. Scale bar: 250 nm. (B) Representative TEM image of cDz. Scale bar: 500 nm. SEM, Scanning Electron Microscopy; TEM, Transmission Electron Microscopy.


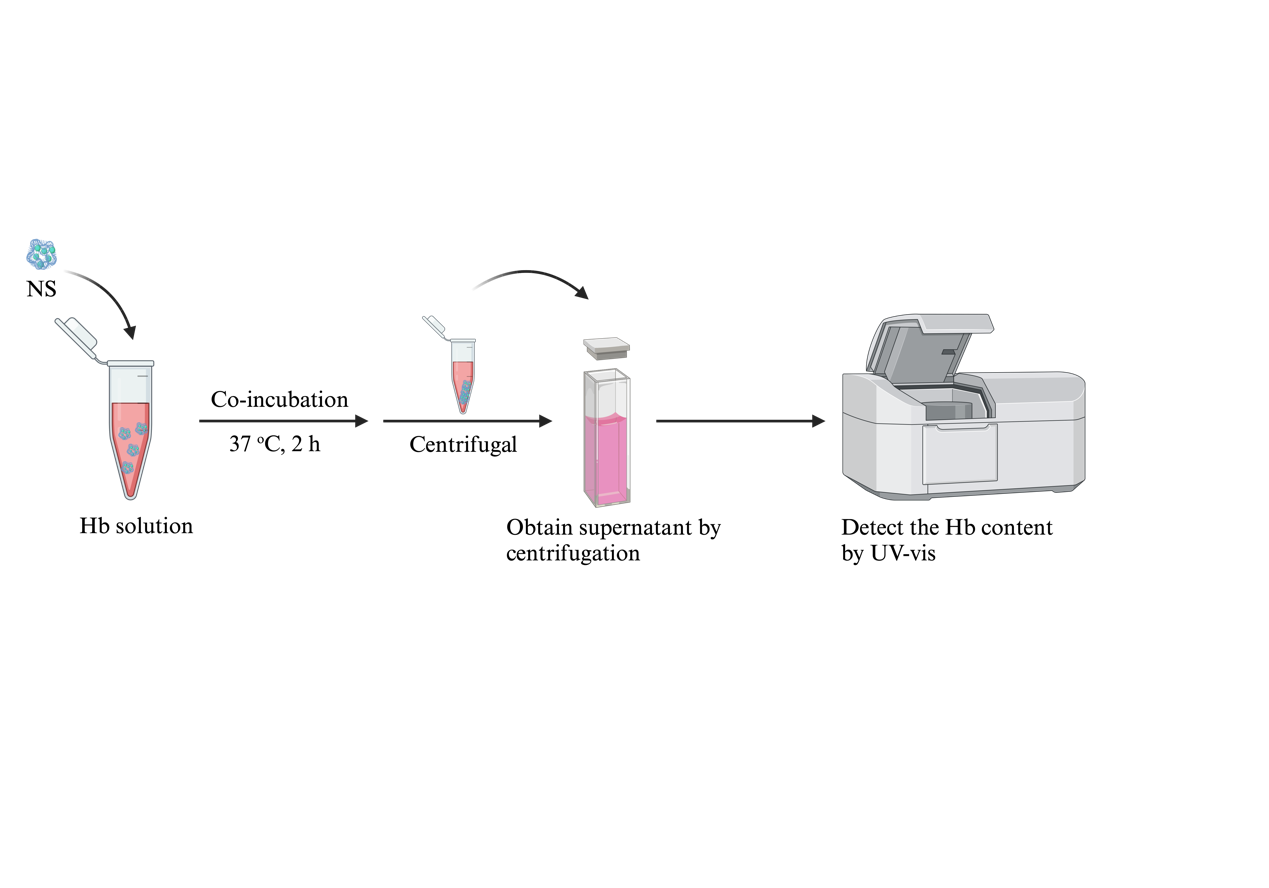


Figure S5. Schematic diagram showing detection of Hb capture with different nanostructures (NS) *in vitro*. Hb, hemoglobin.


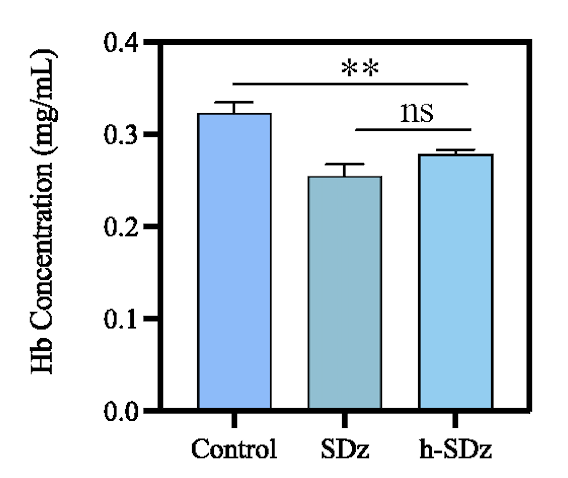


Figure S6. Concentrations of Hb after treatment with SDz or h-SDz (Hb aptamer) (n=3). The results are shown as the mean ± standard deviation. ns, no significant difference, ***P* < 0.01. Statistical significance was calculated using one-way analysis of variance (ANOVA) followed by the post hoc Tukey test. Hb, hemoglobin.


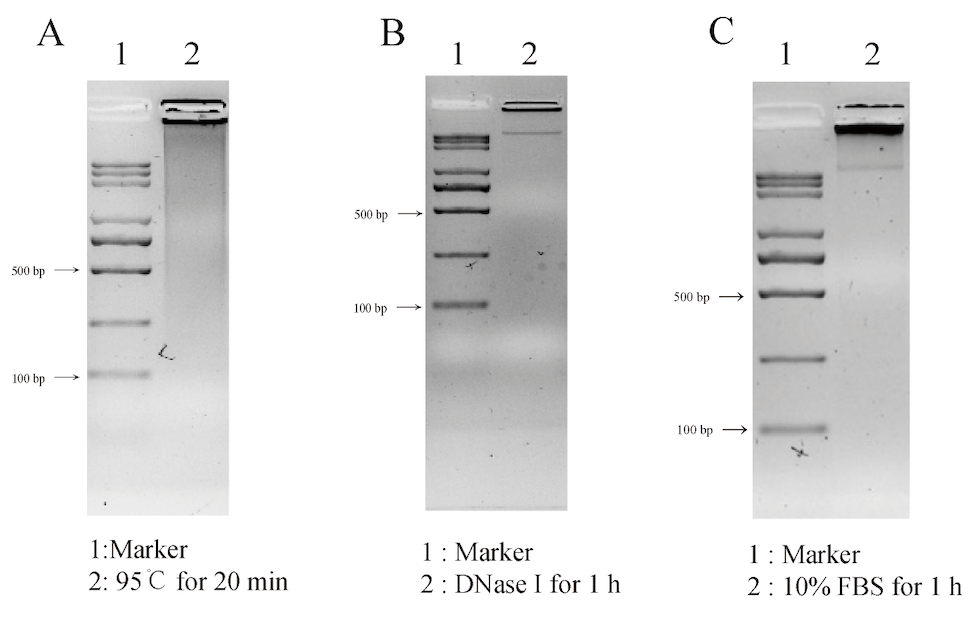


Figure S7. (A) Agarose gel electrophoresis of SDz after treatment at 95℃ for 20 min. (B) Agarose gel electrophoresis of SDz after treatment with 2 U/mL DNase Ⅰ for 1 h. (C) Agarose gel electrophoresis of SDz after treatment with 10% serum for 1 h. FBS, Fetal Bovine Serum.


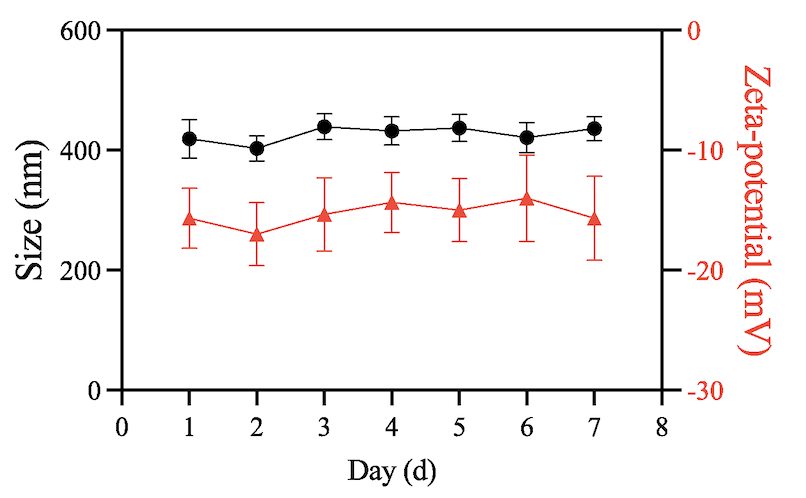


Figure S8. Particle size and zeta potential of SDz in PBS (10 × 10^−3^ M, pH 7.4) at 25℃, sampled at different time points over 7 d (n = 3). PBS, phosphate-buffered saline.


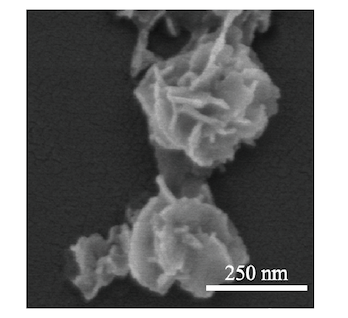


Figure S9. Representative SEM image of SDz after 100-fold dilution and incubation for 1 h. Scale bar: 250 nm. SEM, Scanning Electron Microscopy.


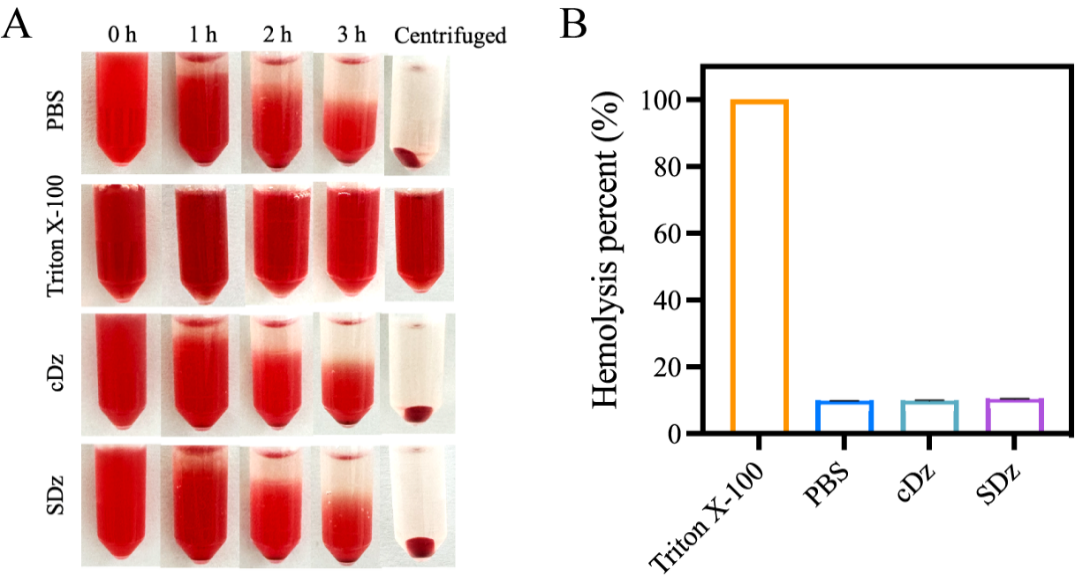


Figure S10. (A) Representative 2% hematocrit images after treatment with cDz or SDz for different lengths of time. (B) Hemolysis percentages after treatment with cDz and SDz (n = 3). The results are shown as the mean ± standard deviation. PBS, phosphate-buffered saline.


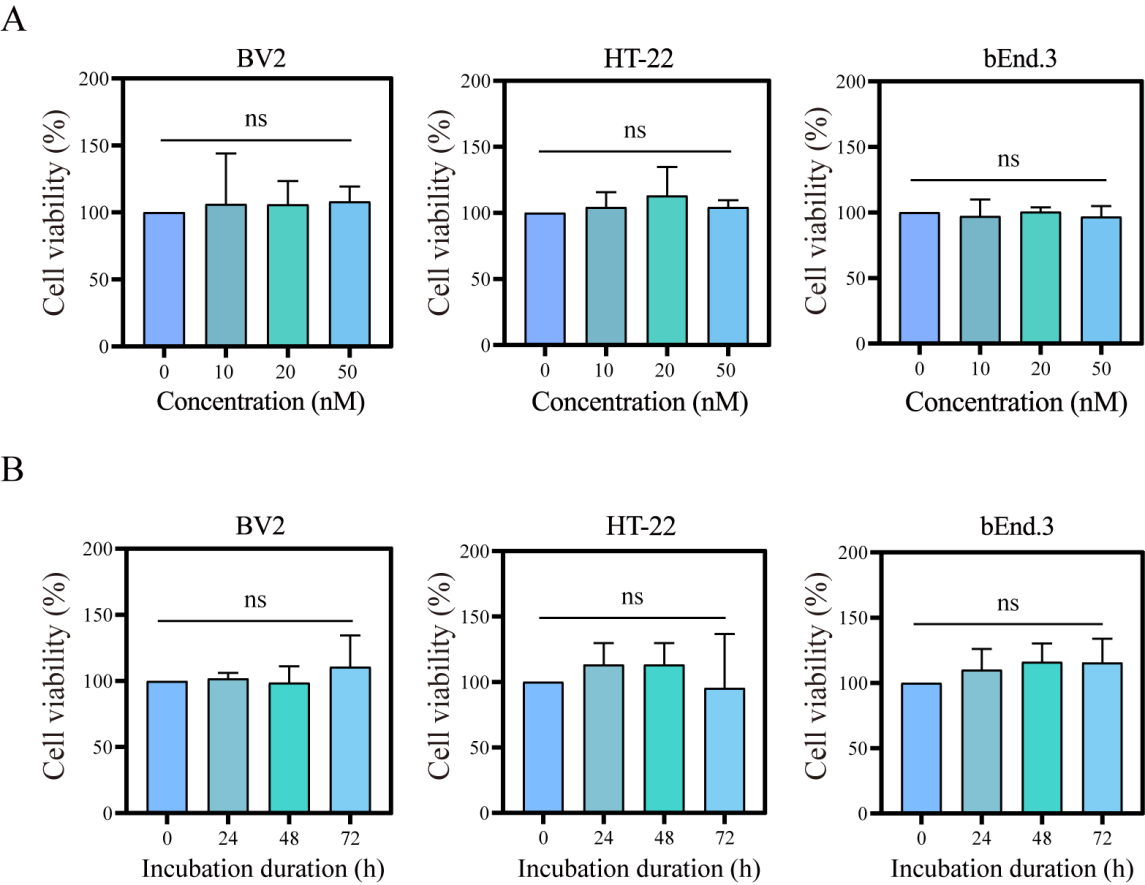


Figure S11. (A) Viability of bEnd.3, BV2, and HT-22 cells after incubation with different concentrations of SDz for 24 h (n=6). (B) Viability of bEnd.3, BV2, and HT-22 cells after incubation with SDz (50 nM) for different durations (n=6). BV2, microglial cells; HT-22, mouse hippocampal neurons; bEnd.3; cerebral vascular endothelial cells. ns, no significant difference. Statistical significance was calculated using one-way analysis of variance (ANOVA) followed by the post hoc Tukey test.


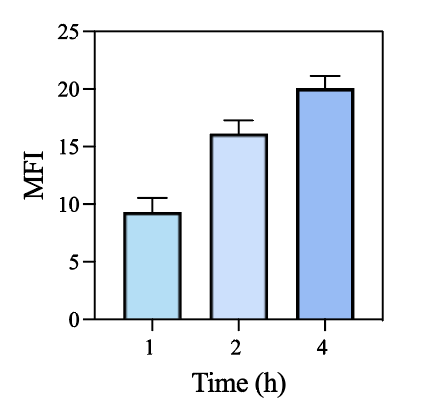


Figure S12. MFI values of Cy5 fluorescence in BV2 cells treated for different lengths of time with SDz to detect cell uptake (n =5). The results are shown as the mean ± standard deviation. MFI, Mean Fluorescence Intensity.


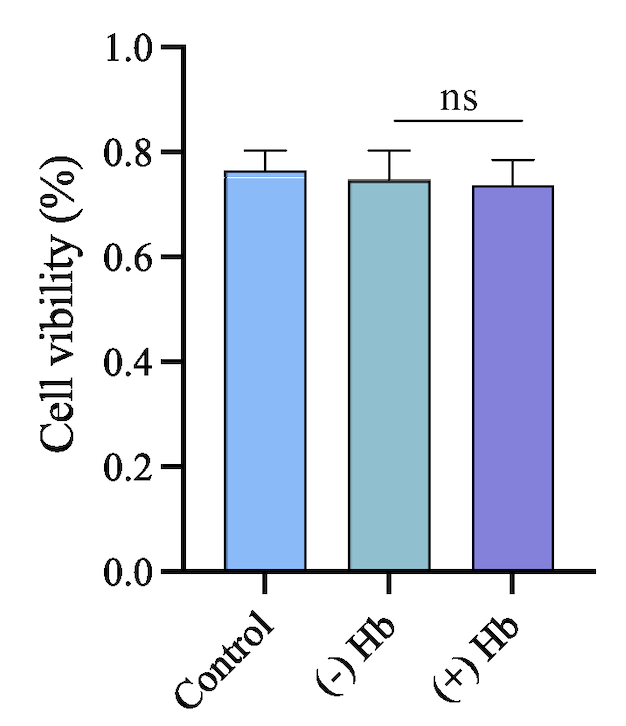


Figure S13. Viability of BV2 cells after treatment with SDz with or without Hb for 24 h. The results are shown as the mean ± standard deviation (n =5). ns, no significant difference. Statistical significance was calculated using one-way analysis of variance (ANOVA) followed by the post hoc Tukey test.


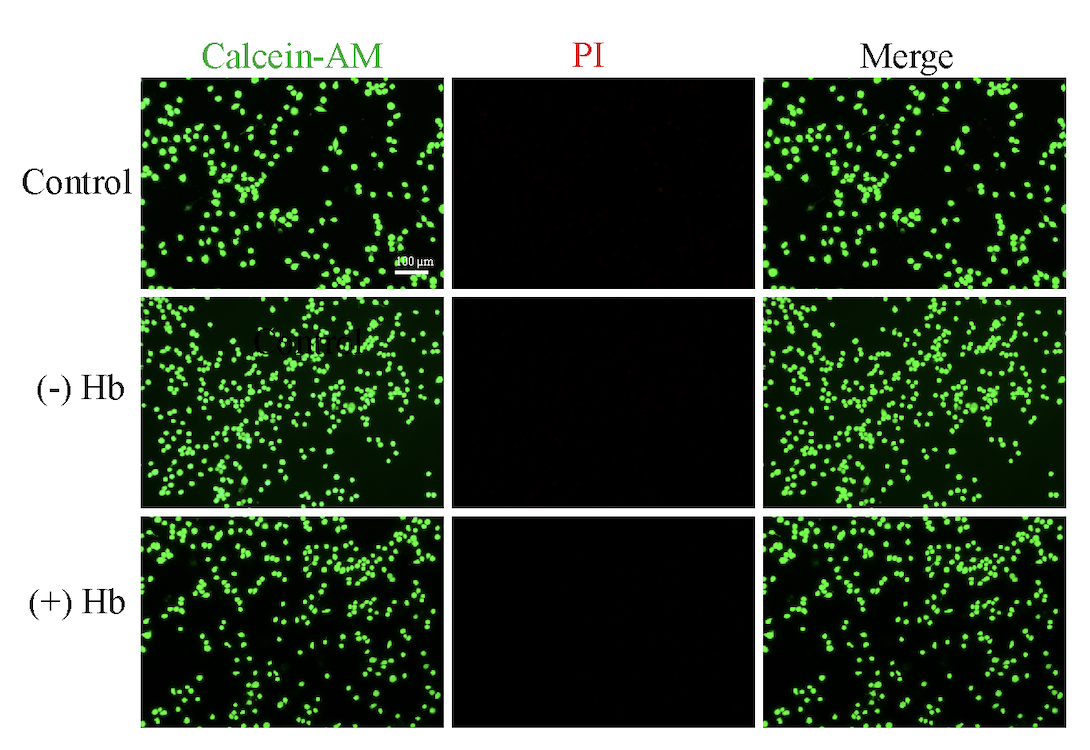


Figure S14. Representative fluorescence images of BV2 cells treated with SDz with or without Hb. Live cells were labeled with Calcein-AM (green), while dead cells were labeled with PI (red). Scale bar: 100 μm. PI, Propidium Iodide.


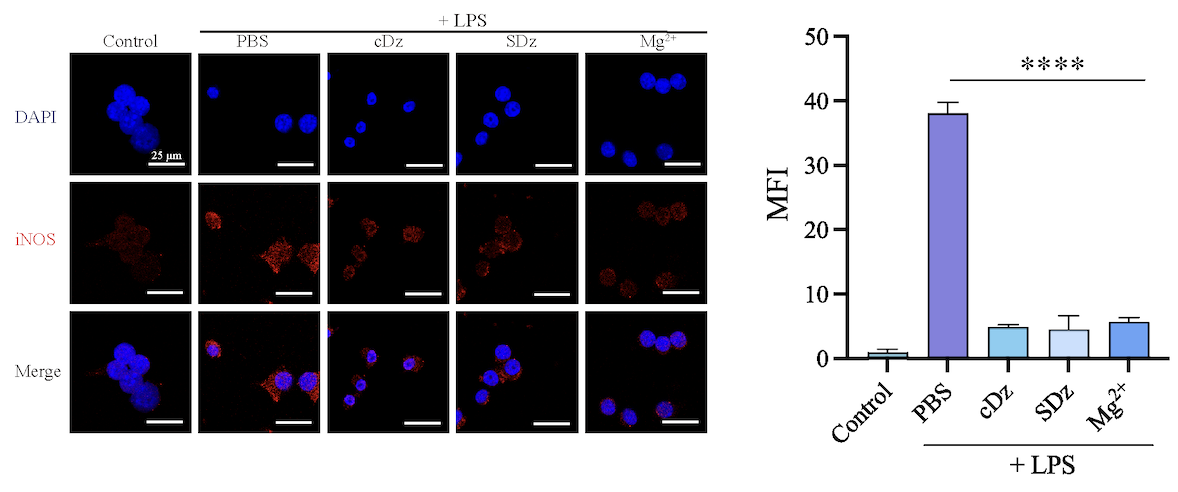


Figure S15. Representative immunostaining images (A) and semi-quantitative analysis (B) of iNOS (red) in BV2 cells (n=3). Scale bar: 25 μm. Data are mean ± standard deviation. *****P* < 0.0001. Statistical analysis was performed using one-way ANOVA followed by Tukey’s HSD post hoc test. LPS, Lipopolysaccharides.


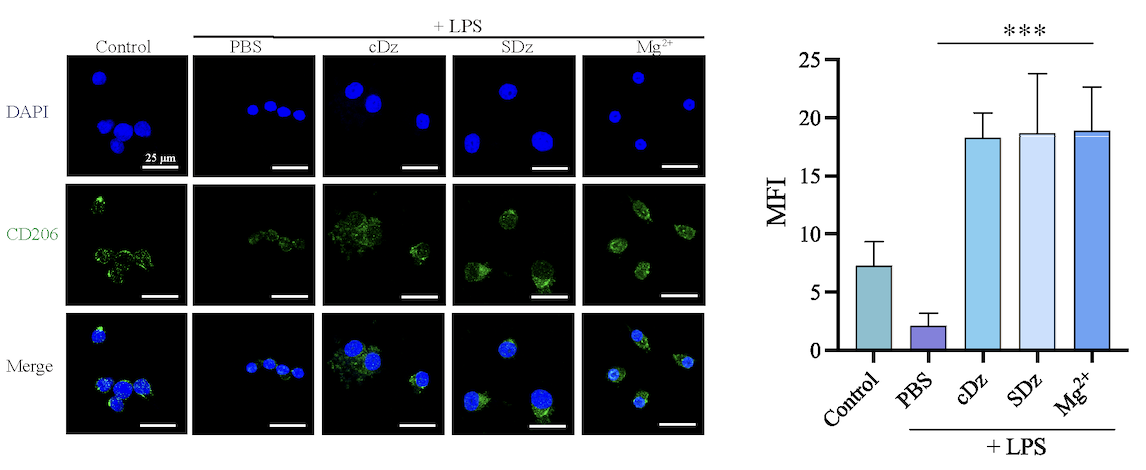


Figure S16. Representative immunostaining images (A) and semi-quantitative analysis (B) of CD206 (green) in BV2 cells. Nuclei were counterstained with DAPI (blue). BV2 cells were induced by incubation with 1 μg/mL^−1^ LPS for 24 h, followed by treatment with PBS, cDz, SDz, or Mg^2+^ (n=3). Scale bar: 25 μm. Data are presented as the mean ± standard deviation. ****P* < 0.001. Statistical analysis was performed using one-way ANOVA followed by Tukey’s HSD post hoc test. LPS, Lipopolysaccharides.


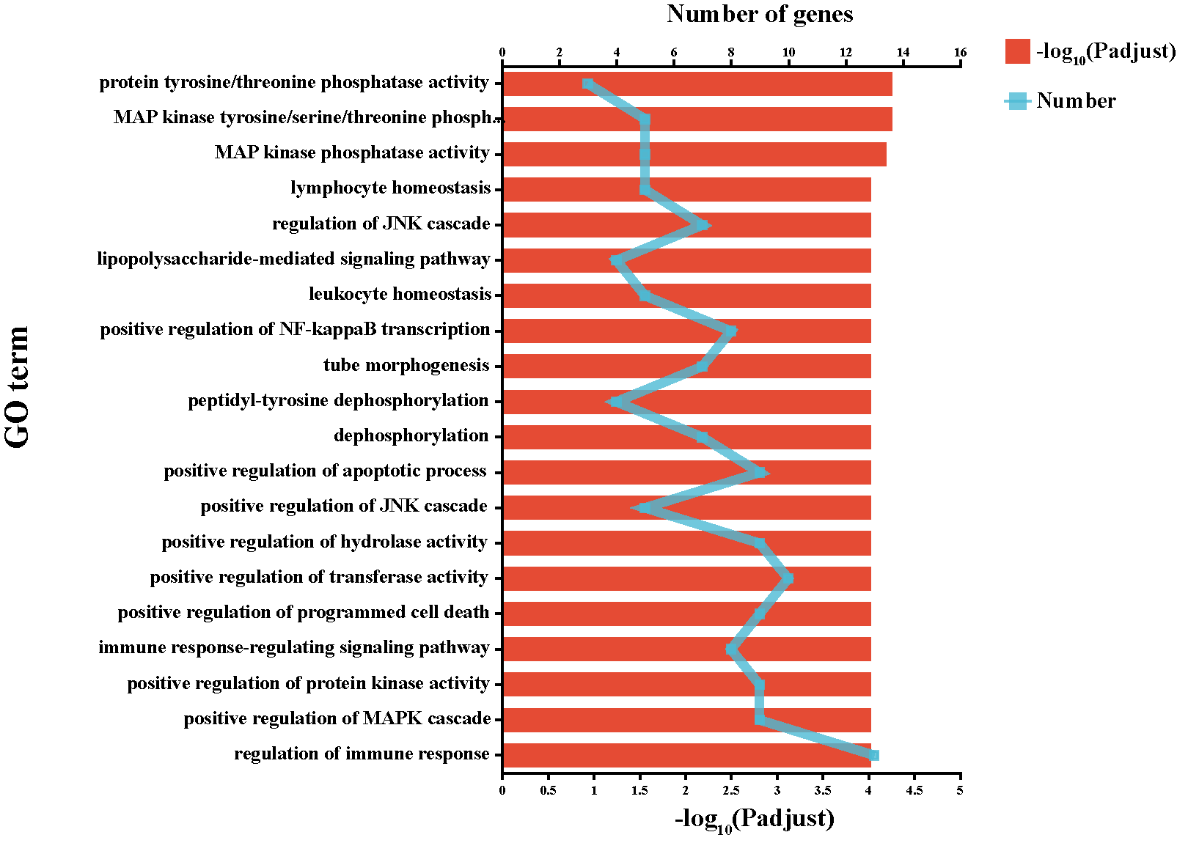


Figure S17. Gene Ontology enrichment of the top 20 up-regulated pathways in the different groups.


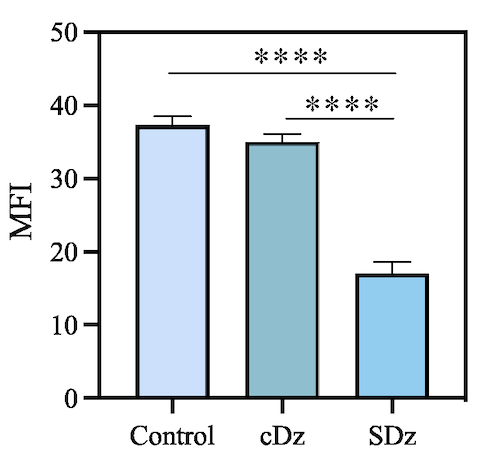


Figure S18. Semi-quantitative immunofluorescence of SIRPα in BV2 cells treated with different nanostructures for 24 h (n=3). The results are shown as the mean ± standard deviation. *****P* < 0.0001. Statistical analysis was performed using one-way ANOVA followed by Tukey’s HSD post hoc test. MFI, Mean Fluorescence Intensity.


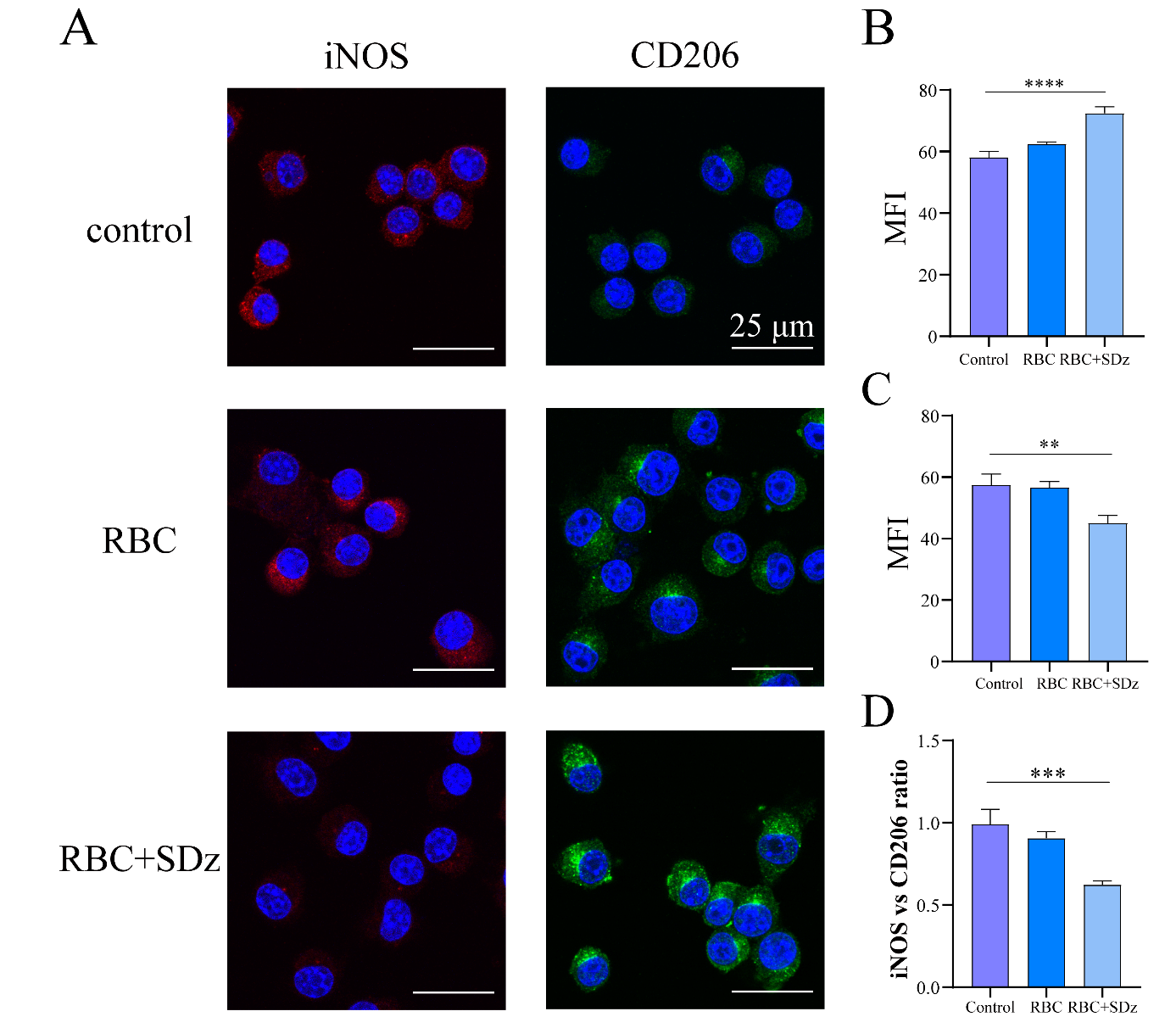


Figure S19. (A) Representative immunostaining images of iNOS (red) and CD206 (green) in BV2 cells (n=3). Semi-quantitative analysis of CD206 (B) and iNOS (C) in BV2 cells (n=3). (D) Expression ratio of iNOS and CD206 , Scale bar: 25 μm. Data are mean ± standard deviation. **P < 0.01, ***P < 0.001, ****P < 0.0001. Statistical analysis was performed using one-way ANOVA followed by Tukey’s HSD post hoc test. RBC, red blood cells; SDz, SIRPα-DNAzyme nanostructures.


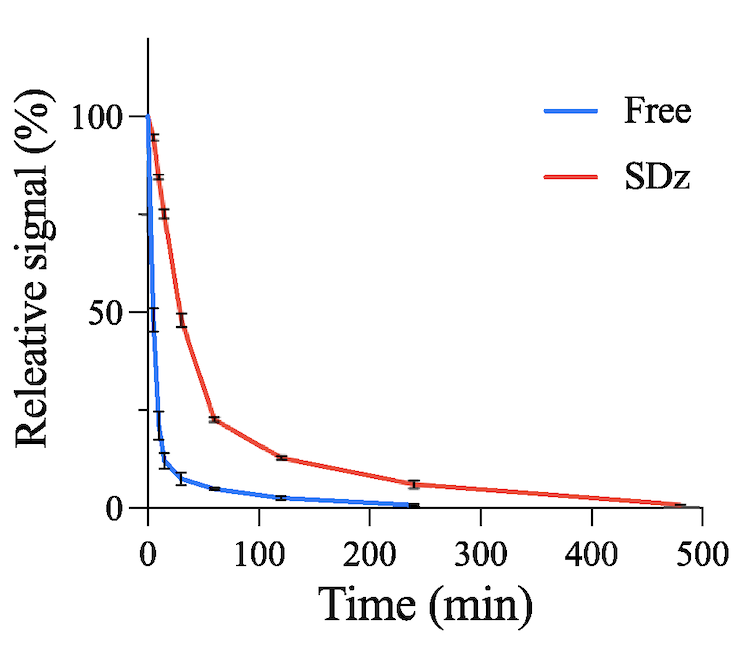


Figure S20. Blood retention of free DNAzyme (Free) and SDz in mice after a single intravenous injection to evaluate the circulation of SDz (n = 3). The results are shown as the mean ± standard deviation.


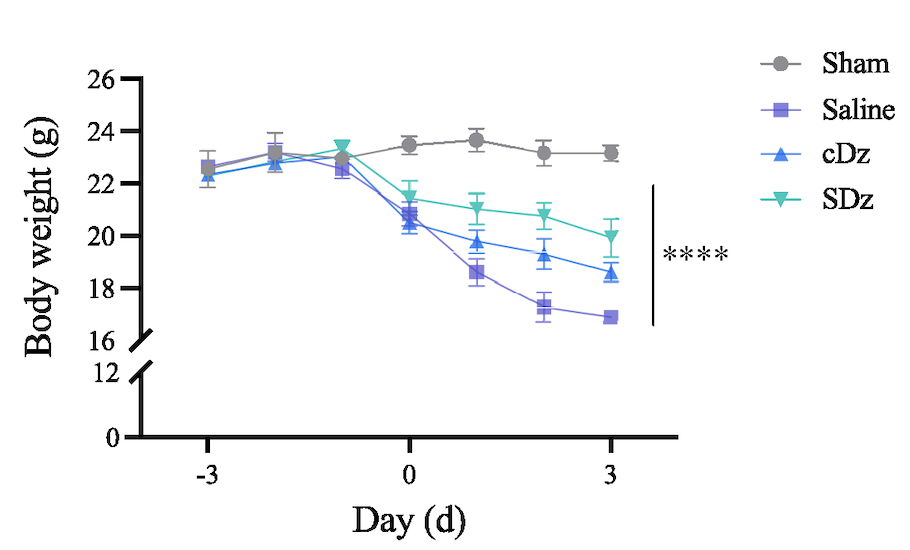


Figure S21. Body weights of mice in the different groups during the whole-animal experiment (n=5). The results are shown as the mean ± standard deviation. *****P* < 0.0001. Statistical analysis was performed using one-way ANOVA followed by Tukey’s HSD post hoc test.


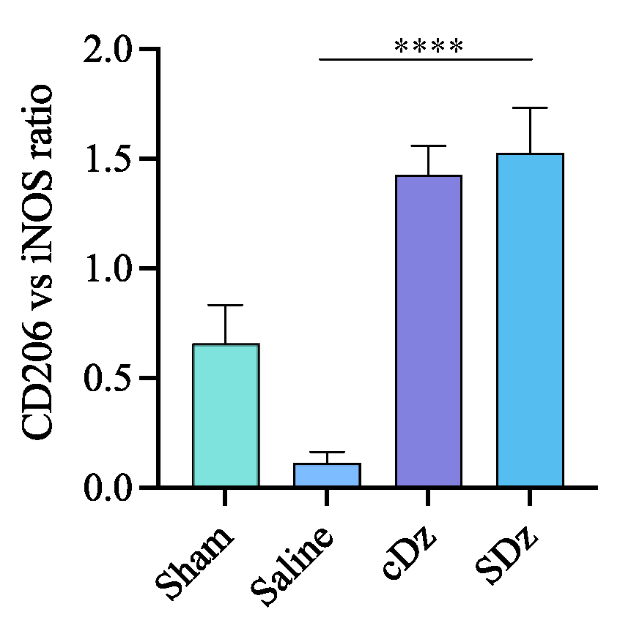


Figure S22. Semi-quantitative immunofluorescence of iNOS and CD206 in mouse brain sections following different treatments (n=3). The results are shown as the mean ± standard deviation. *****P* < 0.0001. Statistical analysis was performed using one-way ANOVA followed by Tukey’s HSD post hoc test.


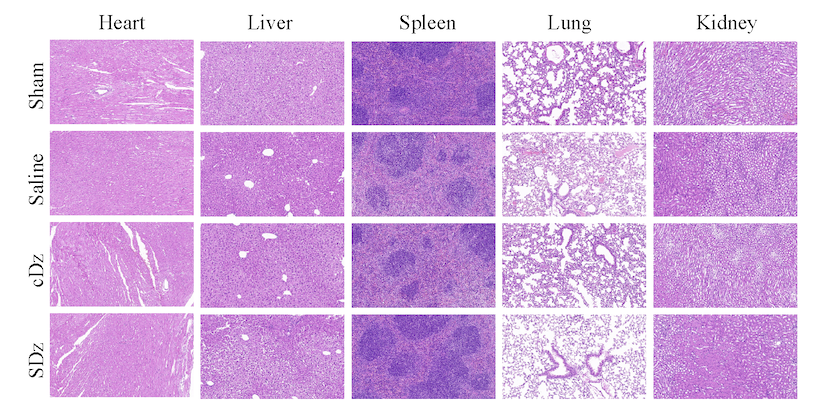


Figure S23. H&E-stained tissue sections of major organs (including the heart, liver, spleen, lungs, and kidneys) from mice following different treatments (n = 3). H&E, hematoxylin, and eosin staining.


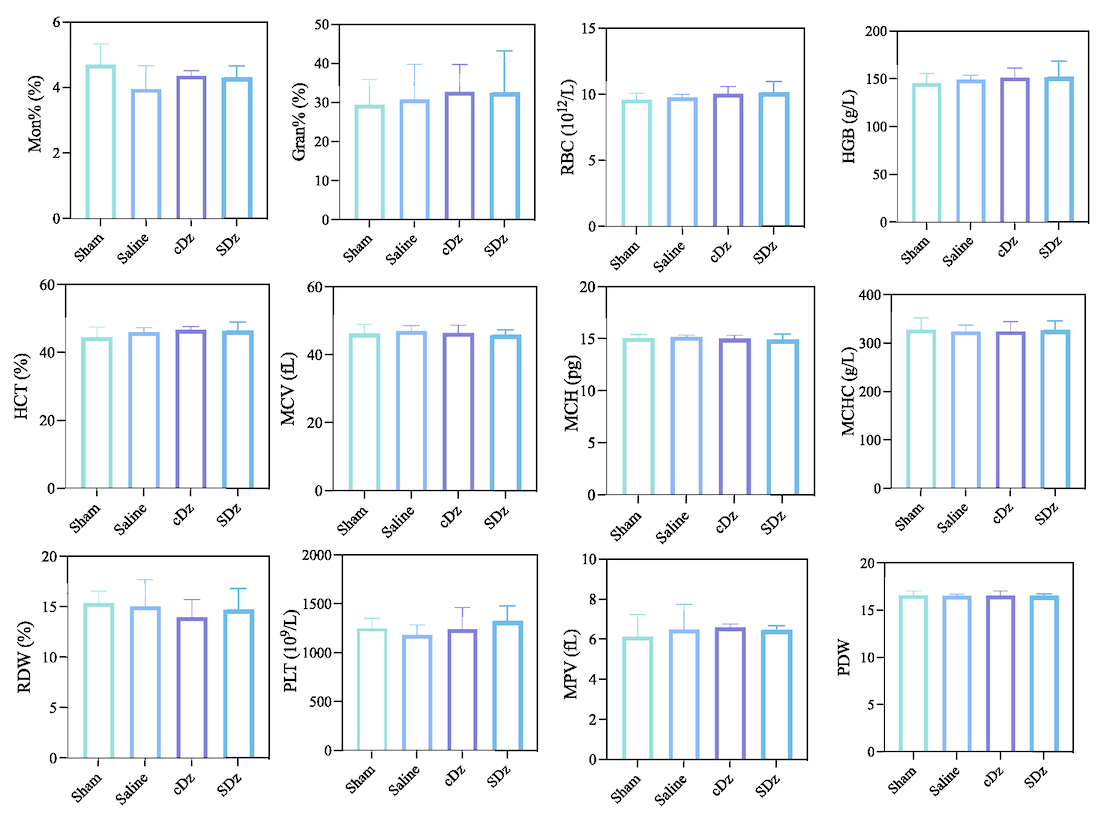


Figure S24. Hematological parameters in mice following different treatments (n = 3). The results are shown as the mean ± standard deviation.


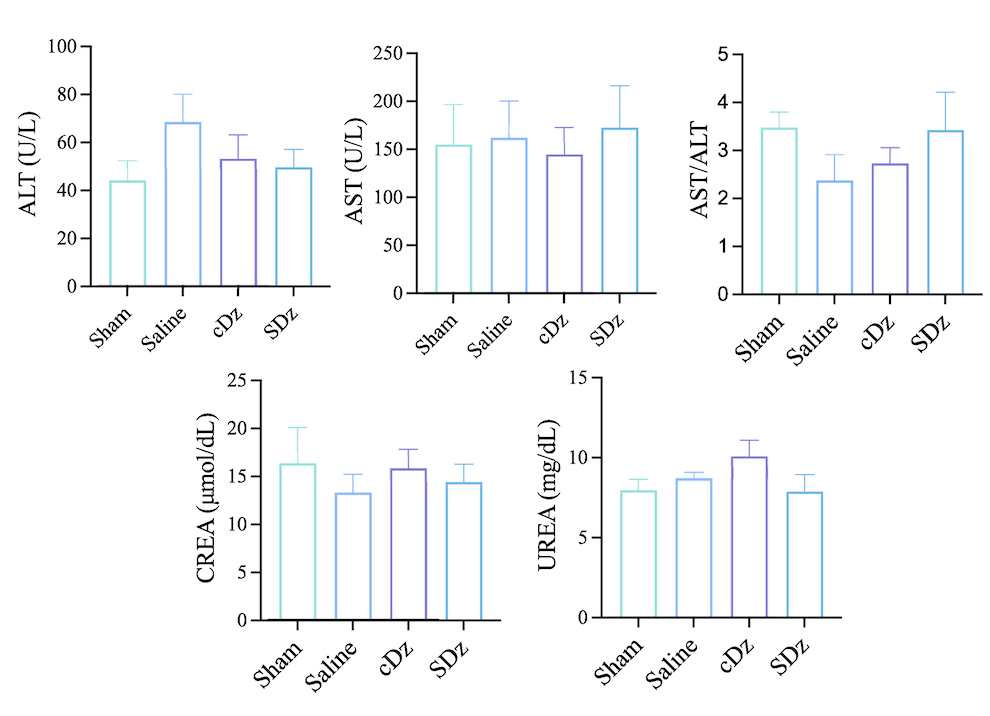


Figure S25. Tests for liver and kidney function in mice following different treatments (n = 3). The results are shown as the mean ± standard deviation.

Table S1. Oligonucleotides used in this study.

| Name | Sequence (5’~3’) |
| --- | --- |
| SIRPα DNAzyme | GAGAGCAGCCAGGCTAGCTACAACGACAGCAGGACTA |
| Substrate mRNA | TAGTCCTGCTG rArU TGGCTGCTCTC |
| Template for DNAzyme | AATAGTCCTGCTGTCGTTGTAGCTAGCCTGGCTGCTCTC AA |
| Primer for DNAzyme | AGCAGGACTATTTTGAGAGCAGCC |
| Template for cDNAzyme | AATAGTCCTGCTGACCTAGGACCATCCGTGGCTGCTCTC AA |
| Primer for cDNAzyme | AGCAGGACTATTTTGAGAGCAGCC |
| Cy5-DNAzyme conjugate | AATAGTCCTGCTGTC |
| Fam-DNAzyme conjugate | CAGCAGGACTA-FAM |
| BHQ1-DNAzyme conjugate | BHQ-GAGAGCAGCCA |
| Cy5-DNAzyme for Magnetic bead experiment | GAGAGCAGCCAGGCTAGCTACAACGACAGCAGGACTA |
| Cy5-Hb-apt for Magnetic bead experiment | ACGCACACCAGAGACAAGTAGCCCCCCAAACGCG |
| mus-GAPDH F primer | GAGAAACCTGCCAAGTATGATGAC |
| mus-GAPDH R primer | AGAGTGGGAGTTGCTGTTGAAG |
| mus-SIRPα F primer | CAACGACATCACATACGCAGAC |
| mus-SIRPα R primer | TGTGGTTGTTAGGCTCAGGG |
